# Incremental predictive value of CMR phenotyping for major adverse cardiovascular events: an interpretable machine learning study from UK Biobank

**DOI:** 10.3389/fmed.2026.1852547

**Published:** 2026-07-31

**Authors:** Peiqi Liu, Chang Liu, Yao Ma, Zhi Lv, Kexin Li, Zi Kou, Pei Wang, Fuxian Ren, Dengfeng Gao

**Affiliations:** 1 Department of Cardiology, The Second Affiliated Hospital of Xi'an Jiaotong University, Xi'an, China; 2 Xi'an Jiaotong University Xi'an, China; 3 Department of Cardiology, Meishan Branch of the Third Affiliated Hospital, Yan'an University School of Medicine, Meishan, Sichuan, China

**Keywords:** cardiac magnetic resonance, machine learning, major adverse cardiovascular events, risk prediction, UK Biobank

## Abstract

**Aims:**

To characterize cardiac magnetic resonance (CMR)-derived phenotypes in a population-based cohort and to evaluate their incremental prognostic value for major adverse cardiovascular events (MACE) beyond traditional risk factors.

**Methods:**

Participants from the UK Biobank imaging cohort without prior cardiovascular diseases were included. Unsupervised k-means clustering and Kaplan–Meier analyses identified distinct CMR-derived phenotypes. Sequential XGBoost models were developed to evaluate the incremental predictive value of CMR features over conventional risk factors, with SHAP used for interpretation. Cross-modality transportability was assessed in an independent Asian cohort using echocardiographic data as a surrogate for CMR-derived variables.

**Results:**

Among 27,254 participants, 785 (2.88%) experienced MACE over a median follow-up of approximately 5 years. Two phenotypes were identified, with the higher-risk phenotype characterized by increased cardiac volumes, impaired cardiac function, a tendency toward myocardial hypertrophy, and reduced myocardial strain. The CMR-enhanced model improved MACE prediction compared with traditional risk factors alone (AUC: 0.76, 95% CI: 0.73–0.79; vs. AUC: 0.61, 95% CI: 0.58–0.64, *P* < 0.001), with strong performance for heart failure (AUC: 0.90, 95% CI: 0.86–0.94). In the cross-modality validation cohort, the simplified model yielded an AUC of 0.69 (95% CI: 0.56–0.83). Higher age, left ventricular mass index, global wall thickness, and lower levels of high-density lipoprotein cholesterol, left atrial ejection fraction, right atrial stroke volume were the key contributors to increased MACE risk.

**Conclusion:**

CMR-derived phenotypes provide incremental prognostic value beyond traditional risk factors and may improve early identification of individuals at elevated cardiovascular risk.

## 1 Introduction

Cardiovascular diseases remain the leading cause of global mortality, with major adverse cardiovascular events (MACE) representing the most critical clinical outcomes (1). Early and accurate identification of individuals at risk is essential for effective prevention and timely intervention. Cardiac magnetic resonance (CMR) imaging has emerged as a powerful modality in cardiac assessment owing to its unique ability to non-invasively evaluate both structure and function (2). While recent studies have highlighted the superior performance of machine learning (ML)–based automated CMR analysis in cardiovascular diseases diagnosis (3, 4), its application in predicting MACE among asymptomatic individuals remains insufficiently explored. Given that CMR-derived quantitative indices provide detailed insights into cardiac pathophysiology and are closely associated with adverse outcomes, they hold considerable promise for enhancing risk prediction and ultimately reducing the global burden of MACE (5).

Existing cardiovascular risk prediction algorithms are largely based on multivariate regression models that incorporate a limited number of established risk factors and typically assume linear relationships with minimal interaction between predictors. For example, the 2010 American College of Cardiology/American Heart Association (ACC/AHA) guideline recommended use of the Framingham Risk Score (6). While such models have provided an important framework for clinical practice, their restrictive assumptions and narrow set of predictors have yielded only modest predictive performance. In contrast, data-driven ML approaches offer the ability to improve prediction by leveraging large-scale data, uncovering novel predictors, and capturing complex, non-linear interactions (7, 8).

Nevertheless, most prior ML-based cardiovascular risk prediction studies have primarily focused on either traditional clinical risk factors or isolated imaging parameters, often with limited methodological scope. In real-world practice, however, imaging provides a highly informative additional source of data beyond routinely available clinical information. Yet, systematic integration of these comprehensive CMR-derived phenotypes with conventional risk factors within ML frameworks remains scarce. Such an approach may enable more accurate and individualized risk stratification by capturing subtle pathophysiological alterations beyond what clinical variables alone can reflect. To address this gap, the present study aimed to identify distinct CMR-derived phenotypic profiles and evaluate their prognostic value for incident MACE using ML-based approaches in a large, event-free cohort at baseline.

## 2 Materials and methods

### 2.1 Study population and outcomes

This study utilized data from the UK Biobank, a large prospective cohort study investigating risk factors for common adult diseases (9). Between 2006 and 2010, the UK Biobank enrolled 502,493 participants aged 40–69 years, collecting comprehensive data including demographic information, clinical features, and CMR imaging data. The UK Biobank protocol is available online (10).

**Figure 1:**
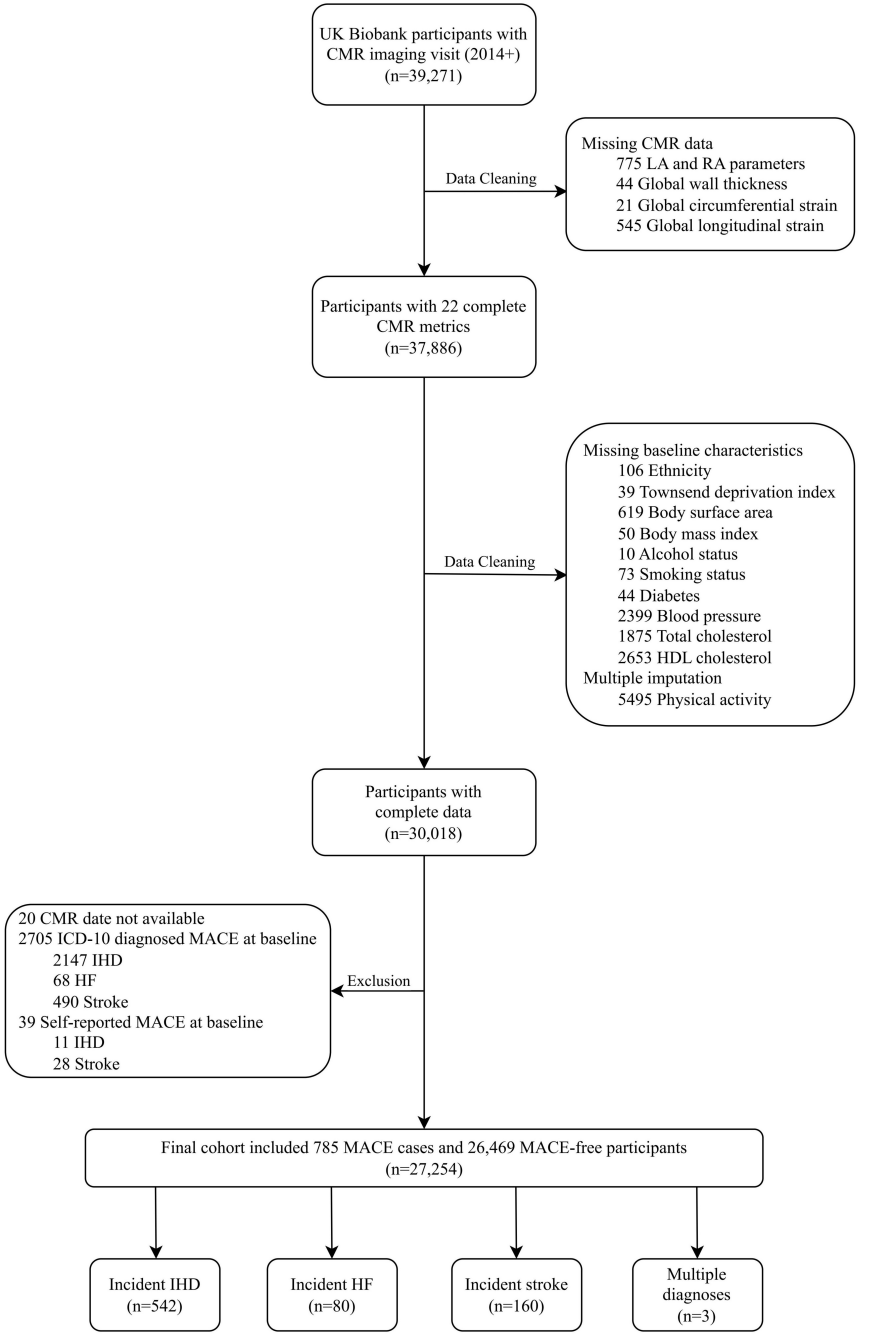
Cohort selection flowchart. Multiple diagnoses: ≥2 MACE events on the same day. CMR, cardiac magnetic resonance; HDL, high-density lipoprotein; HF, heart failure; IHD, ischemic heart disease; ICD, the international classification of diseases; LA, left atrial; MACE, major adverse cardiovascular events; RA, right atrial.

Participants who met the following criteria were included: no prior MACE, including fatal or non-fatal ischemic heart disease (IHD), heart failure (HF), and stroke, as verified through self-report or International Classification of Diseases, 10th Revision (ICD-10) codes (Supplementary Table 1) at the time of CMR examination and availability of CMR data. The participant selection process is illustrated in Figure 1. The primary outcome was MACE, defined as a composite of IHD, HF, or stroke. Secondary outcomes included the individual components of MACE. Incident events were identified using ICD-10 codes (Supplementary Table 1). Follow-up began at the CMR examination date and ended at the earliest of the first occurrence of MACE, death, or October 31, 2022.

### 2.2 Cross-modality transportability cohort

We analyzed an independent cohort of 522 participants identified from the Hospital Information System of the Second Affiliated Hospital of Xi'an Jiaotong University between June 1 2017 and September 1 2025. Eligible participants were free of MACE (IHD, HF, or stroke) at baseline and had available echocardiographic data. Given the limited availability of CMR in resource-constrained settings such as China and other developing nations, echocardiographic data were used as a surrogate for CMR-derived variables. It should be noted that these two imaging modalities differ substantially in measurement precision, parameter definitions, and inter-observer reproducibility; accordingly, this assessment represents a cross-modality transportability evaluation and more of a surrogate validation rather than a direct external validation of the CMR-based model. Echocardiographic parameters were obtained from routine clinical reports assessed by experienced sonographers in accordance with standard practice guidelines. Outcomes were defined consistently with those in the UK Biobank cohort (IHD, HF, and stroke) and were ascertained from hospital medical records using ICD-10 codes (Supplementary Table 1). Follow-up continued until the first occurrence of an event or September 1, 2025, whichever came first.

### 2.3 Clinical risk factors

We included 15 established clinical risk factors for MACE from the UK Biobank, encompassing demographic variables (sex, age, ethnicity, Townsend deprivation index [TDI]), anthropometric measures (body mass index [BMI], body surface area [BSA]), physiological parameters (systolic and diastolic blood pressure [SBP, DBP], heart rate [HR]), biochemical markers (total and high-density lipoprotein [HDL] cholesterol), lifestyle factors (physical activity [PA], smoking, alcohol consumption), and comorbidities (diabetes status defined by ICD-10 codes or self-report) (Supplementary Table 1). A detailed description is included in Supplementary Method 1.

### 2.4 CMR image acquisition and analysis

A detailed description of CMR image acquisition and analysis is included in Supplementary Method 2. Briefly, CMR imaging was performed using a 1.5-Tesla Siemens MAGNETOM Aera scanner (11). Short-axis and long-axis cine images were processed through an automated machine learning (ML) pipeline to extract quantitative cardiac metrics, including left and right ventricular and atrial volumes, function, left ventricular (LV) structure and myocardial strain (12). The pipeline was developed and validated using expert annotations from 32,000 UK Biobank CMR studies (13, 14). A comprehensive set of 22 parameters (Table 1), including cardiac volumes, function, structure, and global strain measures were assessed. Quality control procedures included automated checks and expert cardiologist review, ensuring high data integrity.

**Table 1:** Baseline characteristics of the study cohort stratified by MACE outcome.

Baseline characteristics	All cohort (*n* = 27,254)	MACE (*n* = 785)	MACE-free (*n* = 26,469)	*P*-value
Sex, female	14,493 (53.18%)	269 (34.27%)	14,224 (53.74%)	*P* < 0.001
Age, year	54.52 ± 7.43	58.61 ± 6.71	54.40 ± 7.42	*P* < 0.001
Ethnicity, *n* (%)				0.700
Whites	26,481 (97.16%)	765 (97.45%)	25,716 (97.16%)	
Others	773 (2.84%)	20 (2.55%)	753 (2.84%)	
TDI	−1.94 ± 2.68	−1.89 ± 2.74	−1.94 ± 2.68	0.585
BMI, kg/m^2^	26.42 ± 4.12	27.25 ± 3.80	26.39 ± 4.13	*P* < 0.001
BSA, m^2^	1.86 ± 0.21	1.92 ± 0.21	1.86 ± 0.21	*P* < 0.001
SBP, mmHg	136.42 ± 18.57	143.59 ± 19.39	136.21 ± 18.50	*P* < 0.001
DBP, mmHg	81.28 ± 10.36	83.06 ± 10.33	81.22 ± 10.36	*P* < 0.001
HR, bpm	67.90 ± 10.78	68.47 ± 11.64	67.89 ± 10.75	0.163
TC, mmol/L	5.76 ± 1.06	5.75 ± 1.07	5.76 ± 1.06	0.833
HDL-C, mmol/L	1.49 ± 0.38	1.37 ± 0.35	1.49 ± 0.38	*P* < 0.001
Diabetes, *n* (%)	851 (3.12%)	53 (6.75%)	798 (3.01%)	*P* < 0.001
Alcohol status, *n* (%)				0.478
Never	682 (2.50%)	23 (2.93%)	659 (2.49%)	
Previous	569 (2.09%)	20 (2.55%)	549 (2.07%)	
Current	26,003 (95.41%)	742 (94.52%)	25,261 (95.44%)	
Smoking status, *n* (%)				*P* < 0.001
Never	16,796 (61.63%)	423 (53.89%)	16,373 (61.86%)	
Previous	8,790 (32.25%)	298 (37.96%)	8,492 (32.08%)	
Current	1,668 (6.12%)	64 (8.15%)	1,604 (6.06%)	
Physical activity, *n* (%)				0.588
Low	5,042 (18.50%)	156 (19.87%)	4,886 (18.46%)	
Moderate	11,377 (41.74%)	325 (41.40%)	11,052 (41.75%)	
High	10,835 (39.76%)	304 (38.73%)	10,531 (39.79%)	

Baseline characteristics were presented as means (standard deviation) for continuous variables and counts (percentages) for categorical variables, compared using *t*-tests and chi-square tests respectively.

MACE, major adverse cardiovascular events; TDI, Thomson deprivation index; BMI, body mass index; BSA, body surface area; SBP, systolic blood pressure; DBP, diastolic blood pressure; HR, heart rate; TC, total cholesterol; HDL-C, high-density lipoprotein cholesterol.

### 2.5 Statistical analysis

Analysis was performed for MACE and three secondary outcomes (IHD, HF and stroke). A more detailed description is provided in Supplementary Method 3. Briefly, multivariable Cox regression models identified independent risk factors for MACE. Dose-response relationships were evaluated with restricted cubic splines (RCS). Unsupervised k-means clustering was used to identify CMR phenotypic subgroups, with MACE risk compared using Kaplan-Meier and log-rank tests. XGBoost models evaluated the prognostic value of CMR metrics, with hyperparameter optimization via grid search and cross-validation. Model interpretability was assessed using Shapley Additive Explanations (SHAP), and performance was evaluated with the area under the receiver operating characteristic curve (AUC) with DeLong's test for comparisons. In addition, model calibration was assessed using calibration plots and Brier score, and clinical utility was evaluated by decision curve analysis (DCA). The C-index with 95% confidence intervals derived from 1,000 bootstrap resamples was also calculated to assess time-to-event discrimination. Feature selection for model simplification was performed using Least Absolute Shrinkage and Selection Operator (LASSO) regression and recursive feature elimination (RFE). Detailed methods are provided in the Supplementary Method 3.

All analyses were performed in Python and R, with statistical significance set at *P* < 0.05.

## 3 Results

### 3.1 Baseline characteristics

After CMR quality control, 37,886 participants had complete imaging data. Following further exclusion of those with missing clinical variable (*n* = 7,868), missing CMR scan date (*n* = 20) and prior MACE (*n* = 2,744), a final analytic cohort of 27,254 participants was included in the study (Figure 1).

Among 27,254 participants (53.18% female), 785 (2.88%) developed MACE during a median follow-up of 1,693 days (Table 1). Incident MACE comprised IHD (*n* = 542), HF (*n* = 80), stroke (*n* = 160), and multiple diagnoses (*n* = 3). Compared with participants without MACE, those who developed MACE were older, predominantly male, with higher BMI, SBP, DBP, BSA, and lower HDL-cholesterol. They also exhibited a higher prevalence of diabetes and smoking history.

A total of 27,254 CMR scans were analyzed at baseline (Table 2). Significant differences were observed between the MACE and MACE-free groups (all *P* < 0.001). Those with MACE had larger cardiac chambers, reflected by increased ventricular end-diastolic volumes and end-systolic volumes. Cardiac function metrics were consistently lower, with reduced ejection fraction and LV global function index (LVGFI). Structural measurements showed increased LV mass (LVM), mass index (LVMI), mass-to-volume ratio (LVMVR), and global wall thickness (GWT). LV myocardial strain analysis demonstrated reduced myocardial deformation across all directions in the MACE cohort.

**Table 2:** Comprehensive CMR indices of participants stratified by MACE status.

CMR-derived indices	All cohort (*n* = 27,254)	MACE (*n* = 785)	MACE-free (*n* = 26,469)	*P*-value
Left ventricle				
LVEDV (mL)	147.55 ± 33.24	156.23 ± 38.09	147.29 ± 33.05	*P* < 0.001
LVEDVI (mL/m^2^)	79.15 ± 13.73	81.39 ± 17.04	79.09 ± 13.61	*P* < 0.001
LVESV (mL)	60.06 ± 18.53	66.62 ± 24.38	59.86 ± 18.29	*P* < 0.001
LVESVI (mL/m^2^)	32.11 ± 8.29	34.67 ± 11.96	32.03 ± 8.14	*P* < 0.001
LVSV (mL)	87.49 ± 19.15	89.61 ± 21.12	87.43 ± 19.08	0.004
LVEF (%)	59.67 ± 5.93	57.94 ± 7.49	59.72 ± 5.87	*P* < 0.001
LVGFI (%)	0.48 ± 0.07	0.45 ± 0.08	0.48 ± 0.07	*P* < 0.001
LVM (g)	85.55 ± 21.95	96.42 ± 25.15	85.23 ± 21.77	*P* < 0.001
LVMI (g/m^2^)	45.64 ± 8.41	49.99 ± 10.64	45.51 ± 8.30	*P* < 0.001
LVMVR (g/mL)	0.58 ± 0.08	0.62 ± 0.10	0.58 ± 0.08	*P* < 0.001
GWT (mm)	5.67 ± 0.76	6.10 ± 0.87	5.66 ± 0.76	*P* < 0.001
Right ventricle				
RVEDV (mL)	156.61 ± 37.21	161.81 ± 38.42	156.45 ± 37.16	*P* < 0.001
RVESV (mL)	67.41 ± 21.13	71.43 ± 22.59	67.29 ± 21.08	*P* < 0.001
RVSV (mL)	89.19 ± 20.21	90.38 ± 21.11	89.16 ± 20.18	0.109
RVEF (%)	57.39 ± 6.04	56.28 ± 6.82	57.43 ± 6.02	*P* < 0.001
Left atrium				
LASV (mL)	43.38 ± 11.72	43.44 ± 12.44	43.38 ± 11.70	0.903
LAEF (%)	61.50 ± 8.97	58.30 ± 11.83	61.59 ± 8.86	*P* < 0.001
Right atrium				
RASV (mL)	40.26 ± 13.21	40.36 ± 14.65	40.25 ± 13.17	0.843
RAEF (%)	47.27 ± 9.17	45.60 ± 10.41	47.32 ± 9.12	*P* < 0.001
Cardiac mechanics				
GCS (%)	−22.38 ± 3.29	−21.29 ± 4.15	−22.41 ± 3.25	*P* < 0.001
GRS (%)	45.16 ± 8.18	43.69 ± 9.61	45.21 ± 8.13	*P* < 0.001
GLS (%)	−18.53 ± 2.75	−17.65 ± 3.25	−18.56 ± 2.73	*P* < 0.001

Values are presented as mean ± standard deviation. Group differences are assessed using Student's *t*-test.

CMR, cardiac magnetic resonance; MACE, major adverse cardiovascular events; LVEDV, left ventricular end-diastolic volume; LVEDVI, left ventricular end-diastolic volume index; LVESV, left ventricular end-systolic volume; LVESVI, left ventricular end-systolic volume index; LVSV, left ventricular stroke volume; LVEF, left ventricular ejection fraction; LVGFI, left ventricular global function index; LVM, left ventricular mass; LVMI, left ventricular mass index; LVMVR, left ventricular mass-to-volume ratio; GWT, global wall thickness; RVEDV, right ventricular end-diastolic volume; RVESV, right ventricular end-systolic volume; RVSV, right ventricular stroke volume; RVEF, right ventricular ejection fraction; LASV, left atrial stroke volume; LAEF, left atrial ejection fraction; RASV, right atrial stroke volume; RAEF, right atrial ejection fraction; GCS, global circumferential strain; GRS, global radial strain; GLS, global longitudinal strain.

### 3.2 Independent risk factors and dose-response relationship

Independent risk factors for MACE and specific outcomes were identified using multivariate Cox regression (Supplementary Figure 2). Strong independent risk factors included diabetes (HR: 1.43, 95% CI: 1.06–1.92, *P* = 0.020), current smoking status (HR: 1.32, 95% CI: 1.01–1.72, *P* = 0.044), total cholesterol (HR: 1.08, 95% CI: 1.01–1.16, *P* = 0.032), LV end-systolic volume index (LVESVI, HR: 1.22, 95% CI: 1.08–1.37, *P* = 0.001) and RV ejection fraction (RVEF, HR: 1.07, 95% CI: 1.01–1.12, *P* = 0.015). In contrast, HDL-cholesterol (HR: 0.52, 95% CI: 0.40–0.68, *P* < 0.001) and left atrial ejection fraction (LAEF, HR: 0.98, 95% CI: 0.98–0.99, *P* < 0.001) were inversely associated with risk. Global circumferential strain (GCS, which takes negative values) also showed a protective effect (HR: 1.05, 95% CI: 1.01–1.10, *P* = 0.011). As detailed in Supplementary Figure 3, Cox regression analyses revealed distinct risk factor profiles for secondary outcomes. TDI was identified as an independent risk factor for IHD (HR: 1.04, 95% CI: 1.01–1.07, *P* = 0.020). For HF, RVEF demonstrated a more pronounced association (HR: 1.12, 95% CI: 1.01–1.25, *P* = 0.033). Meanwhile, global radial strain (GRS) emerged as a significant independent predictor for stroke (HR: 1.04, 95% CI: 1.01–1.07, *P* = 0.012).

Based on multivariable Cox regression models, RCS analyses further demonstrated significant U-shaped associations for LVESVI, RVEF, GCS, HDL-cholesterol and an L-shaped relationships for age and LAEF (all *P* < 0.001; Supplementary Figure 2). The lowest risk of MACE was observed at approximately 23.76 mL/m^2^ for LVESVI, 59.43% for RVEF, −22.20% for GCS and 2.08 mmol/L for HDL-cholesterol. For age and LAEF, a pronounced increase in MACE risk was observed beyond the threshold of 50.14 years and 48.22%, respectively.

### 3.3 CMR phenotypes and risk prediction

**Figure 2:**
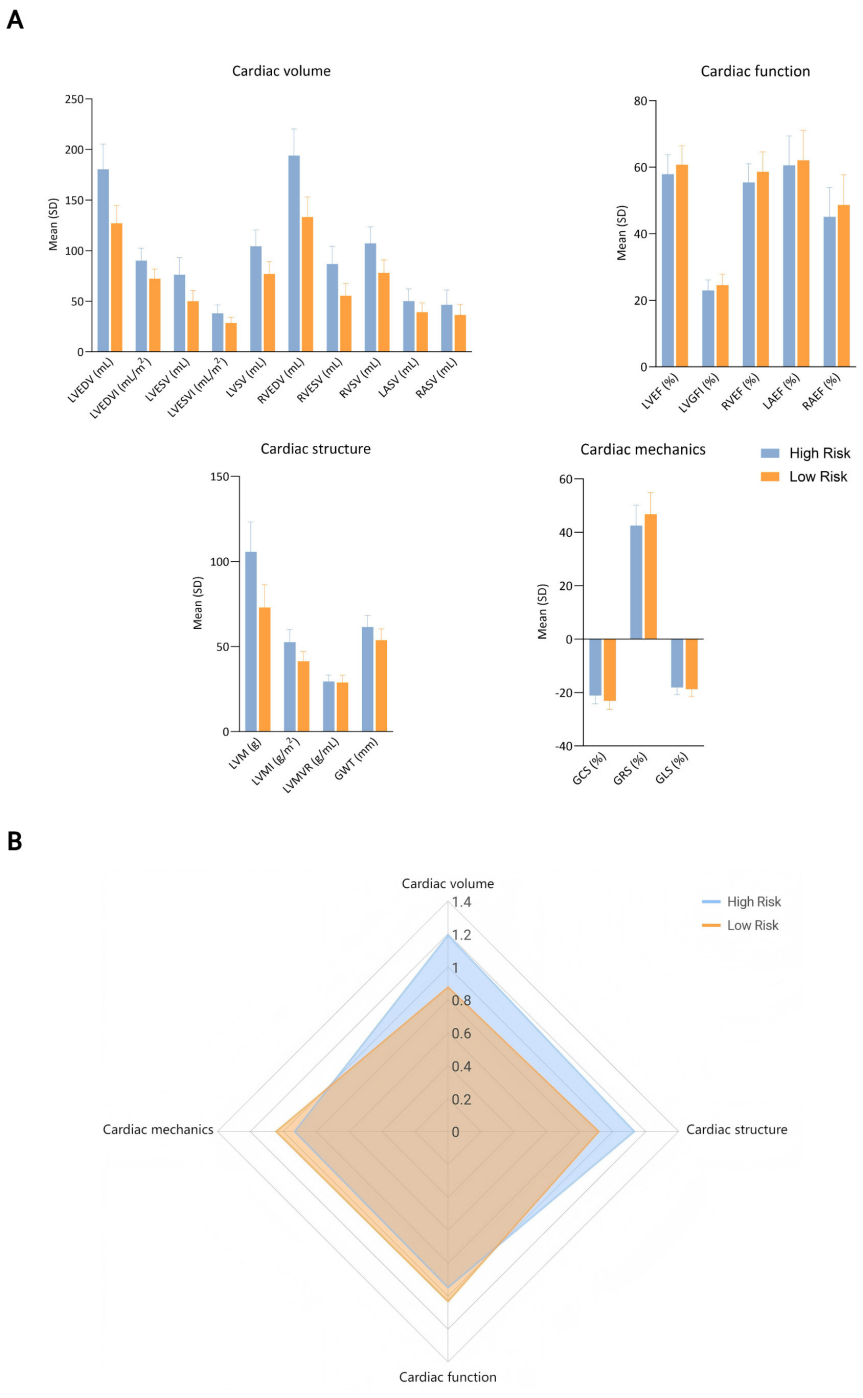
CMR phenotypes identified by k-means clustering analysis. **(A)** Bar plot illustrating differences in CMR metrics between clusters (High-Risk cluster: *n* = 10,481; Low-Risk cluster: *n* = 16,773). The vertical axis represents the mean value of each metric. All intergroup differences remain statistically significant after Bonferroni correction (*p* < 0.001). **(B)** Radar chart comparing phenotypic features between clusters, encompassing cardiac volume, function, structure, and mechanics. To enhance clarity, LVGFI and LVMVR values are scaled by 50, and GWT by 10. Each metric is normalized to the population mean for its respective dimension, and the normalized values within each dimension are summed to reflect the overall contribution of that dimension. CMR, cardiac magnetic resonance; GCS, global circumferential strain; GLS, global longitudinal strain; GRS, global radial strain; GWT, global wall thickness; LASV, left atrial stroke volume; LAEF, left atrial ejection fraction; LVEDV, left ventricular end-diastolic volume; LVEDVI, left ventricular end-diastolic volume index; LVESV, left ventricular end-systolic volume; LVESVI, left ventricular end-systolic volume index; LVGFI, left ventricular global function index; LVM, left ventricular mass; LVMI, left ventricular mass index; LVMVR, left ventricular mass-to-volume ratio; LVEF, left ventricular ejection fraction; LVSV, left ventricular stroke volume; MACE, major adverse cardiovascular events; RASV, right atrial stroke volume; RAEF, right atrial ejection fraction; RVEDV, right ventricular end-diastolic volume; RVESV, right ventricular end-systolic volume; RVSV, right ventricular stroke volume; RVEF, right ventricular ejection fraction.

K-means clustering analysis identified two distinct cardiac phenotypes with significantly different risks of MACE using the elbow method, with a silhouette score of 0.42 indicating moderate-to-good cluster stability (high-risk cluster: *n* = 10,481; low-risk cluster: *n* = 16,773). The high-risk phenotype was characterized by larger cardiac volumes, impaired cardiac function, a tendency toward LV hypertrophy, and reduced myocardial strain (Figure 2).

**Figure 3:**
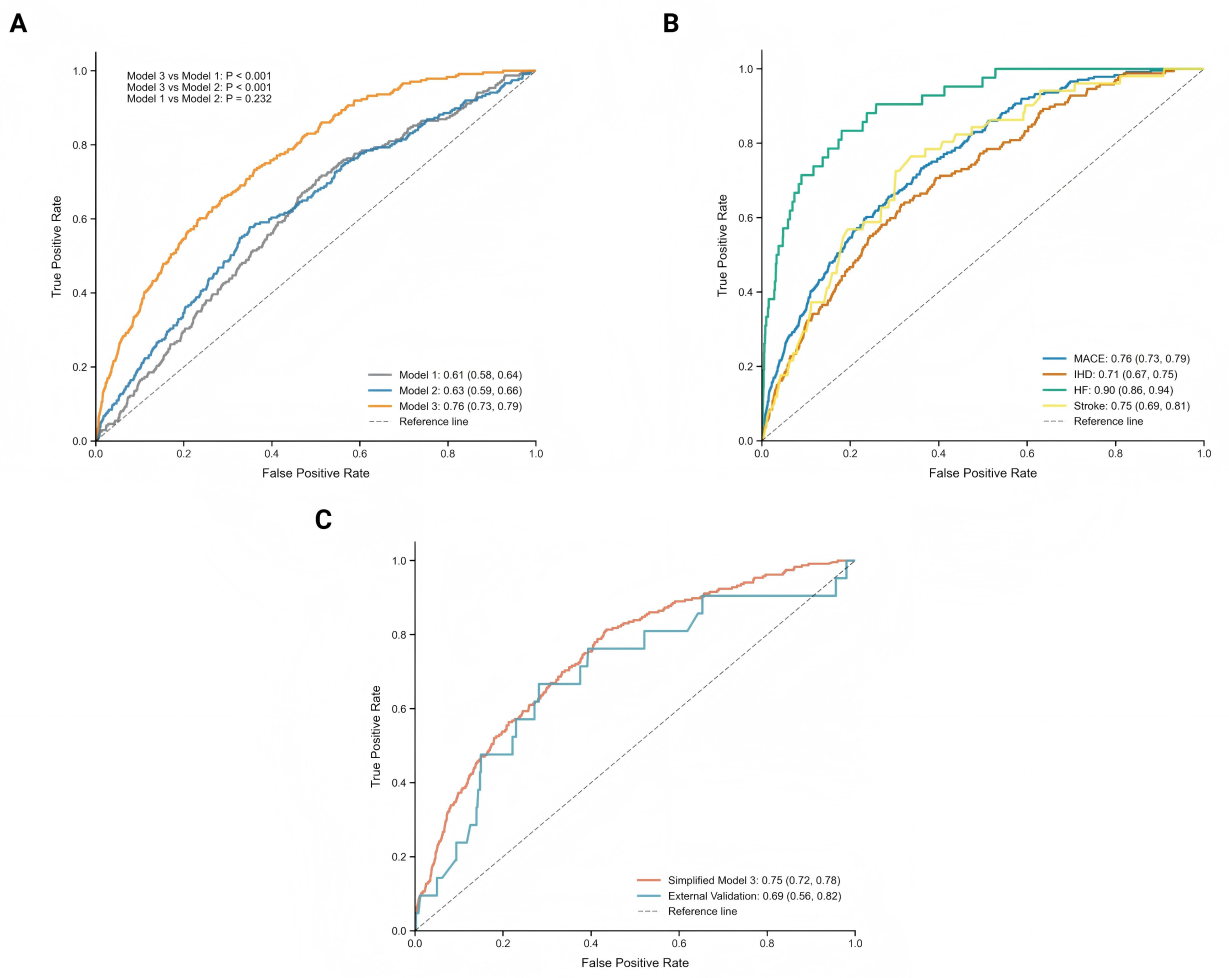
Performance comparison of XGBoost prediction models for MACE. Model 1 included 15 traditional risk factors. Model 2 incorporated three “echocardiographic parameters” (left ventricular end-diastolic volume, left ventricular end-systolic volume, and left ventricular ejection fraction) on top of Model 1. Model 3 further added 22 CMR metrics to Model 1. Simplified Model 3 comprises eight variables including age, body surface area, left ventricular end-diastolic volume index, left ventricular end-systolic volume index, left ventricular ejection fraction, left ventricular global function index, left ventricular mass index and left ventricular mass-to-volume ratio. **(A)** ROC curves of three sequential XGBoost models for predicting MACE risk. **(B)** ROC curves evaluating the performance of the XGBoost models across all MACE outcomes. **(C)** ROC curves of simplified Model 3 and its cross-modality validation. CMR, cardiac magnetic resonance; HF, heart failure; IHD, ischemic heart disease; MACE, major adverse cardiovascular events; ROC, receiver operating characteristic; XGBoost, Extreme Gradient Boosting.

The XGBOOST models, integrating CMR biomarkers and clinical risk factors, demonstrated progressive improvements in predictive performance, underscoring the incremental prognostic value of CMR metrics (Figure 3). Model 1, including only clinical risk factors, achieved an AUC of 0.61 (95% CI: 0.58–0.64) for MACE. Model 2, which added three CMR-derived cardiac structural and functional measures commonly assessed in echocardiography, showed a modest improvement with an AUC of 0.63 (95% CI: 0.59–0.66) for MACE. By contrast, Model 3, incorporating comprehensive CMR metrics, achieved an AUC of 0.76 (95% CI: 0.73–0.79) for MACE and showed consistent and superior discrimination beyond Model 1 and Model 2 across all secondary outcomes (all *P* < 0.001). The highest discrimination was observed for HF (AUC: 0.90, 95% CI: 0.86–0.94; *n* = 80 events) (Supplementary Table 2). Evaluated within a time-to-event framework in the internal validation set, Model 3 maintained robust MACE discrimination with a Harrell's C-index of 0.76 (95% CI: 0.73–0.79).

**Figure 4:**
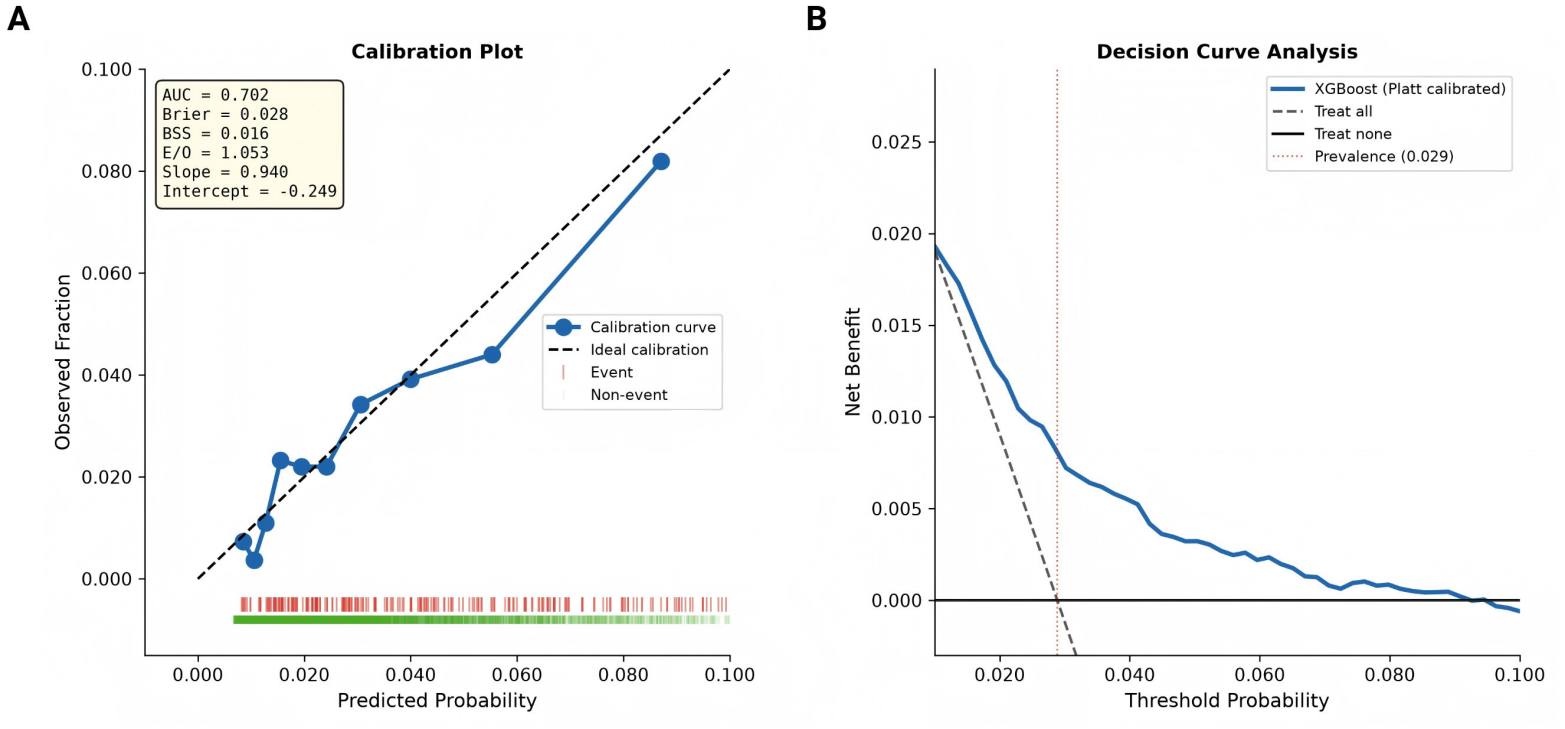
Calibration and decision curve analysis of the optimized XGBoost model for MACE prediction in the internal validation set. **(A)** Calibration plot showing predicted probabilities vs. observed MACE fractions. The dashed diagonal line indicates ideal calibration, and the blue line represents the Platt-calibrated model (metrics detailed in the inset). Red and green rug plots display event and non-event distributions, respectively. **(B)** Decision curve analysis demonstrating net benefit across threshold probabilities. The blue line represents the calibrated model, contrasted with the “treat-all” (gray dashed line) and “treat-none” (black solid line) strategies. The vertical red dotted line denotes the baseline prevalence (0.029). AUC, area under the receiver operating characteristic curve; BSS, Brier skill score; E/O, expected-to-observed; MACE, major adverse cardiovascular events.

In the internal validation cohort, Model 3 also maintained consistent but attenuated performance, with an AUC of 0.70 (95% CI: 0.67–0.73), as expected compared with the development dataset. The model also demonstrated good calibration and overall probabilistic accuracy, with a Brier score of 0.028 and an expected-to-observed (E/O) ratio of 1.053 (95% CI: 0.936–1.197), indicating strong agreement between predicted and observed event rates without evidence of systematic over- or underestimation (Figure 4A). Finally, decision curve analysis (DCA) showed that the model provided a positive net clinical benefit compared with both “treat-all” and “treat-none” strategies across a wide range of clinically relevant threshold probabilities, supporting its potential utility for clinical risk stratification (Figure 4B).

### 3.4 Model interpretability

**Figure 5:**
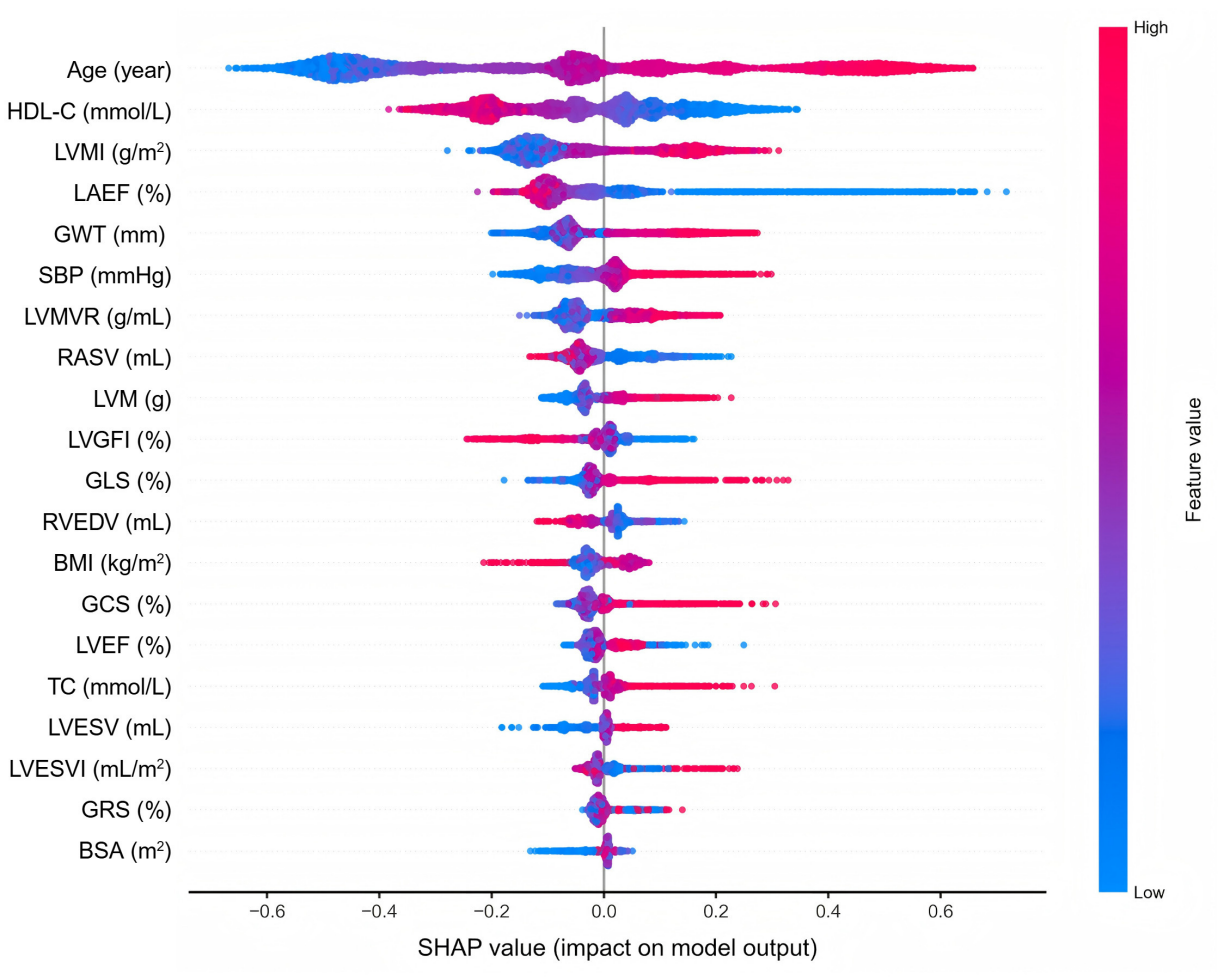
SHAP analysis for MACE. SHAP summary plot displaying the top 20 features influencing MACE prediction, ranked by importance along the y-axis. Each dot represents an individual patient, with color indicating feature value (blue = lower, red = higher). The x-axis shows the SHAP value, reflecting each feature's contribution to the model output; positive values indicate increased likelihood of MACE, and negative values indicate reduced likelihood. Key adverse predictors include age (years), LVMI (g/m^2^), and GWT (mm), where higher values are associated with increased MACE risk, while HDL-cholesterol (mmol/L), LAEF (%), and RASV (mL) are prominent protective features, where higher values are associated with reduced risk. BMI, body mass index; BSA, body surface area; GCS, global circumferential strain; GLS, global longitudinal strain; GRS, global radial strain; GWT, global wall thickness; HDL-C, high-density lipoprotein cholesterol; LAEF, left atrial ejection fraction; LV, left ventricular; LVEF, LV ejection fraction; LVESV, LV end-systolic volume; LVESVI, LV end-systolic volume index; LVGFI, LV global function index; LVM, LV mass; LVMVR, LV mass-to-volume ratio; LVMI, LV mass index; LVEDV, LV end-diastolic volume; MACE, major adverse cardiovascular events; RASV, right atrial stroke volume; RV, right ventricular; RVEDV, RV end-diastolic volume; SBP, systolic blood pressure; SHAP, Shapley Additive Explanations; TC, total cholesterol.

SHAP analysis enhanced the interpretability and transparency of the CMR-based model for MACE by quantifying feature importance and ranking the top predictors (Figure 5). Higher age, LVMI and GWT emerged as the strongest risk factors, whereas elevated HDL-cholesterol, LAEF and right atrial stroke volume (RASV) were identified as the most prominent protective factors against MACE. Notably, age consistently emerged as the most important adverse predictor across all secondary outcomes (Supplementary Figure 5). For IHD, reduced HDL-cholesterol combined with increased LVMI and GWT conferred higher risk. For HF, lower GCS, left ventricular ejection fraction (LVEF) and LAEF were the strongest risk factors. For stroke, reduced global longitudinal strain (GLS) and LVGFI, together with elevated GWT and LVM were associated with greater risk.

SHAP dependence plots illustrated how variations in feature values, in interaction with other variables, influenced predictions and revealed critical inflection points in risk trajectories (Supplementary Figure 6). Specifically, MACE risk increased markedly when key predictors exceeded approximate exploratory cut-off values, including age >55 years, LVMI >50 g/m^2^, and GWT >6 mm. Conversely, risk appeared lower when LAEF exceeded 50% and HDL-cholesterol was above 1.40 mmol/L, as well as when RASV was greater than 30 mL. We further utilized SHAP dependence plots to explore inflection points for the independent risk factors identified by multivariate Cox regression, including LVESVI of approximately 30 mL/m^2^ and GCS of approximately −23%, broadly consistent with RCS-derived estimates. Subsequent waterfall plots visualized individual cardiovascular risk profiles by decomposing the positive and negative contributions of personal biomarkers (Supplementary Figure 7).

### 3.5 Model simplification and cross-modality validation

To enhance clinical applicability while preserving predictive accuracy, we simplified the CMR-based Model 3 by retaining the ten most influential predictors and subsequently evaluated its transportability in an independent echocardiography-derived cohort. Considering the limited availability of echocardiographic variables, one non-echocardiographic predictor (right ventricular end-diastolic volume) was first excluded. As LV end-diastolic volume (LVEDV) and LV end-diastolic volume index (LVEDVI) contributed comparably (Supplementary Figure 8), LVEDV was excluded to mitigate multicollinearity, whereas LVEDVI was retained. The final model therefore comprised eight predictors: age, BSA, LVEDVI, LVESVI, LVEF, LVMI, LVMVR, and LVGFI. The simplified model achieved an AUC of 0.75 (95% CI: 0.72–0.78) in the UK Biobank cohort (Figure 3).

In the independent echocardiography-derived cohort, 21 participants (4.02%) experienced MACE during a median follow-up of 253 days (Supplementary Table 3). Cross-modality assessment in this external dataset yielded an AUC of 0.69 (95% CI: 0.56–0.83).

## 4 Discussion

In this large-scale cohort, CMR phenotyping of subclinical myocardial remodeling improved risk stratification for MACE beyond traditional risk factors. Using both conventional models and machine learning, we confirmed the incremental prognostic value of CMR, supporting its role in precision risk prediction and early preventive strategies.

The incidence of MACE was significantly linked to baseline clinical characteristics and CMR metrics, suggesting the potential value of integrating imaging with traditional risk assessment. Notably, the markedly higher prevalence of diabetes in MACE individuals underscoring its potential role in driving adverse myocardial remodeling. This finding aligns with previous studies documenting both structural and functional cardiac alterations in diabetic populations (15, 16), suggesting that diabetes-associated cardiac remodeling may precede overt MACE, highlighting the importance of early detection of subclinical myocardial dysfunction in diabetic populations.

Multivariable Cox regression combined with RCS analysis identified several independent risk factors for MACE and highlighted important nuances in the associations between cardiac measurements and MACE risk. Notably, diabetes and current smoking remained strong contributors to adverse outcomes, underscoring the complex interplay between metabolic disorders, unhealthy lifestyle behaviors, and the development of MACE. These findings reinforce the need for targeted prevention and enhanced health education strategies. The observed association between increased LVESVI and elevated MACE risk supported the notion of LV subclinical remodeling as an early marker of disease progression (17). Regarding RVEF, although participants with MACE had lower mean RVEF than those without (56.28% vs. 57.43%, *P* < 0.001), multivariable Cox regression identified RVEF as an independent risk factor with HR >1, likely reflecting confounding in the unadjusted comparison. RCS analysis further revealed a U-shaped relationship, suggesting that both reduced and elevated RVEF confer increased risk, consistent with prior studies indicating that deviations from the normal range may reflect maladaptive cardiac responses to subclinical cardiac stress or adverse loading conditions (18, 19).

The combination of advanced cardiac imaging with data-driven unsupervised clustering analysis enhanced the detection of intrinsic phenotypic patterns in complex clinical datasets, which reinforced the robustness of CMR imaging biomarkers and supported the potential utility of data-driven CMR assessment in risk prediction. The XGBoost prediction models, combined with interpretable SHAP analysis, validated our findings through a data-driven approach and underscored the incremental prognostic value of CMR metrics. In contrast to prior studies that focused predominantly on clinical features (20, 21), or isolated imaging metrics such as LVM (22), cardiac volumes (23), cardiac function (24), or myocardial strain (25), our study expanded this scope by incorporating a comprehensive array of CMR metrics. This approach enabled a more thorough evaluation of the predictive potential of CMR in MACE. Our XGBoost framework demonstrated stepwise improvement in predictive performance across outcomes, with the highest discrimination observed for HF. This may reflect the sensitivity of CMR-derived measures in capturing structural and functional cardiac alterations relevant to HF pathophysiology. However, given the relatively small number of HF events, this finding should be considered exploratory and may be influenced by outcome imbalance or model instability. Replication in larger, independent cohorts is warranted to confirm the robustness of this finding. Importantly, beyond discrimination performance, the model also demonstrated favorable overall performance across multiple complementary evaluation dimensions in the internal validation set. Within a time-to-event framework, the Harrell's C-index of 0.76 confirmed consistent and acceptable discriminative ability. Furthermore, the model exhibited good calibration and overall probabilistic accuracy. Although the calibration intercept (−0.249) suggested a slight baseline overestimation of absolute risk, the Platt-calibration effectively mitigated this imbalance, as evidenced by a near-ideal E/O ratio (1.053) and a low Brier score (0.0276), confirming generally accurate risk predictions in this low-prevalence setting (2.88%). Finally, while the decision curve analysis demonstrated a positive net clinical benefit compared with both “treat-all” and “treat-none” strategies across a range of threshold probabilities, this finding should be interpreted with caution, as the ability to detect meaningful clinical net benefit is inherently limited in low-prevalence settings. Therefore, although the model shows promise for risk stratification, its incremental clinical benefit in decision-making may be limited in low-prevalence populations and should be validated in larger and more event-rich cohorts.

SHAP analysis further underscored the interplay between conventional risk factors and advanced CMR-derived features, highlighting their complementary value in refining cardiovascular risk assessment. Notably, SHAP dependence plots revealed exploratory inflection points for age, LAEF, LVESVI, GCS, and HDL-cholesterol that closely corresponded to those identified in the RCS analysis, providing complementary support for non-linear associations. Previous studies have demonstrated the prognostic value of LAEF, LVESVI and GCS for cardiovascular events (25–27). The present analysis extends these findings by providing additional characterization of non-linear risk trajectories, highlighting the added interpretability provided by SHAP.

To explore the applicability of the simplified model beyond the UK Biobank setting, we performed a cross-modality transportability assessment in an independent Asian cohort using echocardiographic data, reflecting real-world settings where CMR may be less accessible. Although several structural and functional cardiac parameters are physiologically related across CMR and echocardiography, the two modalities differ in image acquisition, measurement precision, reproducibility, and parameter definitions, which may influence model performance. The lower AUC and wider confidence interval observed in the echocardiography-derived cohort likely reflect these methodological differences, together with the limited sample size and follow-up duration. Therefore, the findings should be interpreted as preliminary evidence of cross-modality transportability rather than definitive validation. Further evaluation in larger multi-center cohorts with CMR data is warranted.

In summary, we conducted a large-scale study integrating clinical and CMR imaging-derived biomarkers to develop multi-approach predictive models for MACE. Clustering analysis identified subclinical myocardial alterations among high-risk individuals. The application of interpretable ML identified imaging and clinical features associated with cardiovascular risk and provided insight into their relative contributions to model predictions. Meanwhile, several limitations should be acknowledged. First, the UK Biobank consists predominantly of relatively healthy White individuals, which may limit the representativeness of the primary analysis, and the median follow-up of 5 years precludes direct comparison with established 10-year risk scores such as the Framingham Risk Score, SCORE2, or the PREVENT equations. Second, the study did not account for dynamic changes in CMR parameters. Our models relied on a single time point of CMR indices, whereas longitudinal changes may provide more accurate prognostic information. Third, subgroup analyses were not performed. Given the relatively low incidence of MACE in the UK Biobank, we were unable to assess model performance demographic or clinical strata such as sex, age, or diabetes status. Fourth, external assessment was limited to a small single-center Asian cohort with relatively few events and short follow-up, using echocardiographic rather than CMR data. Differences between these imaging modalities in measurement precision, parameter definitions, and reproducibility may have influenced model performance. The wide confidence interval of the external AUC further highlights the uncertainty of these findings. Larger multi-center prospective studies with CMR-based validation are needed to further evaluate the model's transportability and generalizability. Finally, clinical implementation has not yet been achieved. Our models have not been tested as decision-support tools within healthcare systems, and their feasibility, usability, and stability in real-world practice remain to be established.

Looking forward, future studies could leverage longitudinal CMR follow-up data to explore the temporal dynamics of myocardial remodeling and enhance predictive modeling of MACE. Incorporating serial imaging would allow the evaluation of phenotypic changes over time, potentially identifying early indicators of disease progression or treatment response. Furthermore, integrating multi-omics data, such as genomics and metabolomics, with imaging and clinical features may uncover novel mechanistic pathways and refine individualized risk prediction. Finally, prospective validation in larger and more diverse populations, alongside pragmatic assessments of cost-effectiveness and clinical utility, will be essential to translate CMR-guided risk stratification into routine cardiovascular care.

## 5 Conclusion

In conclusion, integrating CMR phenotypes with machine learning enhances MACE risk prediction beyond traditional clinical factors, supporting individualized risk assessment and precision prevention.

## Data Availability

The original contributions presented in the study are included in the article/Supplementary material, further inquiries can be directed to the corresponding author.
