# Supplementary material for: Incremental predictive value of CMR phenotyping for major adverse cardiovascular events: an interpretable machine learning study from UK Biobank
Source: Front Med (Lausanne). 2026 Jul 31;13:1852547. doi: 10.3389/fmed.2026.1852547 (PMC13472935; doi:10.3389/fmed.2026.1852547)
Supplement: Supplementary file 1 [file Data_Sheet_1.pdf]

Supplementary Material

1 Supplementary Data

1.1 Supplementary Method 1. Baseline clinical risk factors

We analyzed 15 well-established clinical risk factors for major adverse cardiovascular events (MACE), all of which were derived from the UK Biobank resource. These variables were selected based on prior literature on cardiovascular risk evaluation and included demographic, anthropometric, physiological, biochemical, lifestyle, and comorbidity-related factors (1-4).

Demographic characteristics included sex, age, and ethnicity, which were self-reported at baseline. Socioeconomic status was quantified using the Townsend Deprivation Index (TDI), a validated area-based measure of material deprivation derived from participants' residential postal codes, incorporating information on unemployment, car ownership, household overcrowding, and housing status. Anthropometric measurements encompassed body mass index (BMI), calculated as weight in kilograms divided by height in meters squared, body surface area (BSA), estimated using the Mosteller formula, which is widely applied in cardiovascular imaging research. Physiological parameters comprised resting systolic blood pressure (SBP), diastolic blood pressure (DBP), and heart rate (HR), which were measured in a standardized fashion using an automated digital sphygmomanometer (Omron Healthcare) after participants were seated at rest. Heart rate was simultaneously recorded via electrocardiogram (ECG). Biochemical parameters included total cholesterol and high-density lipoprotein (HDL) cholesterol, quantified using enzymatic assays on automated clinical chemistry analyzers, following rigorous internal and external quality control procedures. Non-fasting blood samples were collected at baseline assessment centers (5). Comorbidities such as diabetes status was determined based on either hospital episode statistics coded by the International Classification of Diseases, 10th Revision (ICD-10) (Supplementary Table 1) or self-reported physician diagnosis at baseline. Lifestyle factors, for example, physical activity (PA), smoking behavior, and alcohol consumption were assessed through a standardized touchscreen questionnaire administered at the baseline assessment visit. Physical activity was evaluated using questions adapted from the International Physical Activity Questionnaire (IPAQ), while alcohol and smoking data were categorized into current, previous, or never use. A full description of the UKB assessment protocols is publicly available (https://www.ukbiobank.ac.uk/wp-content/uploads/2025/01/Main-study-protocol.pdf).

1.2 Supplementary Method 2. CMR image acquisition and analysis

CMR imaging was conducted using 1.5-Tesla scanners (MAGNETOM Aera, Syngo Platform VD13A, Siemens Healthcare) (6). Short-axis and long-axis cine images were processed through an automated ML pipeline to extract quantitative cardiac metrics from the left and right ventricles and atria (7). This pipeline was developed with expert manual annotations from approximately 5,000 UK Biobank studies and validated in a large sample of 32,000 UK Biobank CMR studies. Multiple validation studies have demonstrated that ML-based CMR data extraction achieves high accuracy (8-9). The UK Biobank CMR protocol and data access procedures are detailed in reference (10) and raw data can be accessed through the UK Biobank application portal (http://www.ukbiobank.ac.uk/register-apply).

This study included a comprehensive set of 22 parameters: left ventricular (LV) volumes and function measures such as LV end-diastolic volume (LVEDV), LV end-systolic volume (LVESV), LV stroke volume (LVSV), LV ejection fraction (LVEF), LV mass (LVM), LV mass-to-volume ratio (LVMVR) and LV global function index (LVGFI). LVEDV, LVESV and LVM were indexed to BSA to adjust for individual variability, as previously described (11). Right ventricular (RV) volumes indices (RVEDV, RVESV and RVSV) and RV ejection fraction (RVEF), as well as left atrial stroke volume (LASV), right atrial stroke volume (RASV) and atrial ejection fractions (LAEF and RAEF) were also quantified. Myocardial structure was further characterized by global wall thickness (GWT), calculated as the mean thickness across 16 American Heart Association (AHA) segments at LV end-diastole. LV myocardial strain analyses were performed using automated tissue tracking (CVI42, Circle Cardiovascular Imaging Inc), with global longitudinal strain (GLS) derived from long-axis images, and global circumferential strain (GCS) and global radial strain (GRS) obtained from short-axis cine images via non-rigid image registration techniques. These metrics were used to assess cardiac mechanics and calculated as the average peak strain values across 16 AHA segments containing tracked deformation.

Quality control was rigorously implemented, combining automated checks within the analysis pipeline and subsequent expert cardiologist review. Cases with poor image quality, failed segmentation, insufficient cardiac coverage, or physiologically implausible values were excluded. Participants with failed or outlier strain measurements were excluded from strain-specific analyses. After quality control, cardiac imaging phenotypes were available for 39,271 UK Biobank participants. For this study, only individuals with complete CMR data across 22 cardiac parameters were included to ensure data integrity. 37,886 participants met the inclusion criteria and constituted the final analysis cohort (Supplementary Figure 1). Thus, the final study population comprised participants with comprehensive, high-quality CMR datasets.

1.3 Supplementary Method 3. Statistical analysis

Analysis was performed for MACE and three secondary outcomes (IHD, HF and stroke). Baseline variables with <10% missing data were excluded. Missing physical activity data (14.50%) were imputed using multiple imputation by chained equations (MICE) with an ordinal logistic regression model, incorporating all other baseline characteristics as predictors. Five datasets were generated with ten iterations each to ensure stability, and one dataset was selected for the final analysis (Supplementary Figure 2).

Multivariable Cox regression models were constructed to identify independent risk factors for MACE, with hazard ratios (HRs) and 95% confidence intervals (CIs) reported. Continuous variables were standardized for comparability, and the proportional hazards assumption was verified using Schoenfeld residuals. Dose-response relationships between CMR predictors and incident MACE were evaluated using restricted cubic splines (RCS) with knots at the 5th, 35th, 65th, and 95th percentiles, adjusting for clinical covariates (12).

Unsupervised k-means clustering was conducted to identify distinct subgroups and reveal clinically relevant CMR phenotypic patterns. The optimal number of clusters was determined using the elbow method based on within-cluster sum of squares and the silhouette score was used to evaluate cluster stability. Kaplan-Meier analysis and log-rank tests were employed to compare MACE risk across clusters, while inter-cluster differences in CMR metrics were assessed using ANOVA with Bonferroni correction for multiple comparisons.

Three sequential XGBOOST models were developed to assess the incremental prognostic value of CMR metrics: (1) clinical risk factors, (2) clinical risk factors plus three standard CMR metrics (LVEDV, LVESV, and LVEF), and (3) clinical risk factors combined with all available CMR metrics. Given the high cost of CMR in many developing countries, echocardiography remains the primary tool for cardiac assessment. As echocardiographic data are not available in the UK Biobank, three standard CMR-derived measures, commonly evaluated by echocardiography, were used as proxies in this study (13-14). Models were trained with a 70:30 split, incorporating five-fold cross-validation and grid search for hyperparameter optimization. The grid search was conducted over the following ranges: learning rate ∈ {0.01, 0.05, 0.1}, max depth ∈ {1, 2, 3, 5, 7}, n_estimators ∈ {50, 100, 150, 200}, subsample ∈ {0.4, 0.6, 0.8}, colsample_bytree ∈ {0.6, 0.8, 1.0}. Final parameters for MACE prediction included: colsample_bytree = 1.0, learning rate = 0.05, max depth = 3, n_estimators = 50, scale_pos_weight = 10, and subsample = 0.4. Model interpretability was evaluated using SHapley Additive exPlanations (SHAP), with summary plots ranking the top predictors and dependence plots identifying risk thresholds. Discriminative performance was assessed using AUC with bootstrapped 95% CIs (1000 iterations), and pairwise AUC differences were tested with DeLong's test (15), applying Bonferroni correction (significance threshold $p < 0.017$). Model calibration was evaluated using calibration plots and Brier score, and clinical utility was assessed by decision curve analysis (DCA). Additionally, the C-index with 95% confidence intervals derived from 1000 bootstrap resamples was calculated to evaluate time-to-event discrimination. To identify the most relevant predictors for MACE and simplify predictive model, we performed a two-step feature selection procedure. First, cross-validated Least Absolute Shrinkage and Selection Operator (LASSO) regression was applied to all candidate variables to remove those with negligible contributions, retaining only features with non-zero coefficients. For LASSO-based feature selection, all input variables were standardized (mean = 0, SD = 1) prior to model fitting to ensure comparability of penalization across features with different units and scales. Subsequently, recursive feature elimination (RFE) was conducted on the LASSO-selected features, using LASSO as the base estimator, to further refine the model and retain the ten most important predictors for downstream analyses. The resulting refined model was then externally validated in the independent Asian cohort.

All analyses were performed using Python 3.12.4 and R 4.3.2. Statistical significance was defined as a two-sided P value < 0.05, with Bonferroni correction applied for multiple testing where appropriate.

1.4 Supplemental approval file

西安交通大学第二附属医院
医学伦理委员会审查批件

伦审批件号：2025 伦审 228

项目名称	评估基于英国生物银行数据的主要不良心血管事件预测模型在中国人群中的准确性		
项目来源	研究者发起	项目类别	临床~~新技术~~
承担科室	心血管内科	主要研究者	高登峰
审查类别	审查方式	审查时间	审查地点
初始审查	简易程序审查	2025 年 10 月 21 日	NA
审查目录	详见附件 1：医学伦理委员会审查文件目录		
审查委员	程妍		
审查意见	经审查相关材料，该项目符合医学伦理学要求，同意该项目开展。<br>自本伦理委员会审批同意之日起开展该项试验，要求研究者及申办方严格遵守相关法律和法规及临床试验方案，最大限度保护受试者权益和安全。试验过程中应做到：<br>修订方案、知情同意书等文件，更新研究者手册时应提交伦理审查。<br>自审查之日起，一年内尚未完成者，应提交年度/跟踪审查报告。<br>年度定期跟踪审查频率（自审查之日起）：12 个月<br>任何严重不良事件（尤其关注 SUSAR 事件）请立即上报；违反试验方案、暂停/终止试验时应及时提交伦理审查。<br>主任委员签名（盖章）：<br>2025 年 10 月 21 日		
声明	本伦理委员会组成及审批过程均按照《赫尔辛基宣言》、《涉及人的生物医学研究伦理审查办法》、《药物临床试验质量管理规范》、《医疗器械临床试验质量管理规范》等法规操作。		

医学伦理委员会联系地址：西安市新城区皇城西路 30 号　　联系电话：029-87678326

附件 1：

西安交通大学第二附属医院

医学伦理委员会审查文件目录

项目名称	评估基于英国生物银行数据的主要不良心血管事件预测模型在中国人群中的准确性	
主要研究者	心血管内科　高登峰	

编号	文件名称	版本号/批号/效期/日期
1	科学性审查结论	
2	伦理审查申请表	
3	研究人员信息	
4	研究方案	版本号：1.0　日期：2025.09.15
5	豁免知情同意声明	
6	研究材料诚信与研究者责任声明	
7	研究经费来源证明	
8	无利益冲突声明	
9	研究成果发布形式说明	

西安交通大学第二附属医院医学伦理委员会

第二附属医院医学伦理委员会

2 Supplementary Figures and Tables

2.1 Supplementary Figures

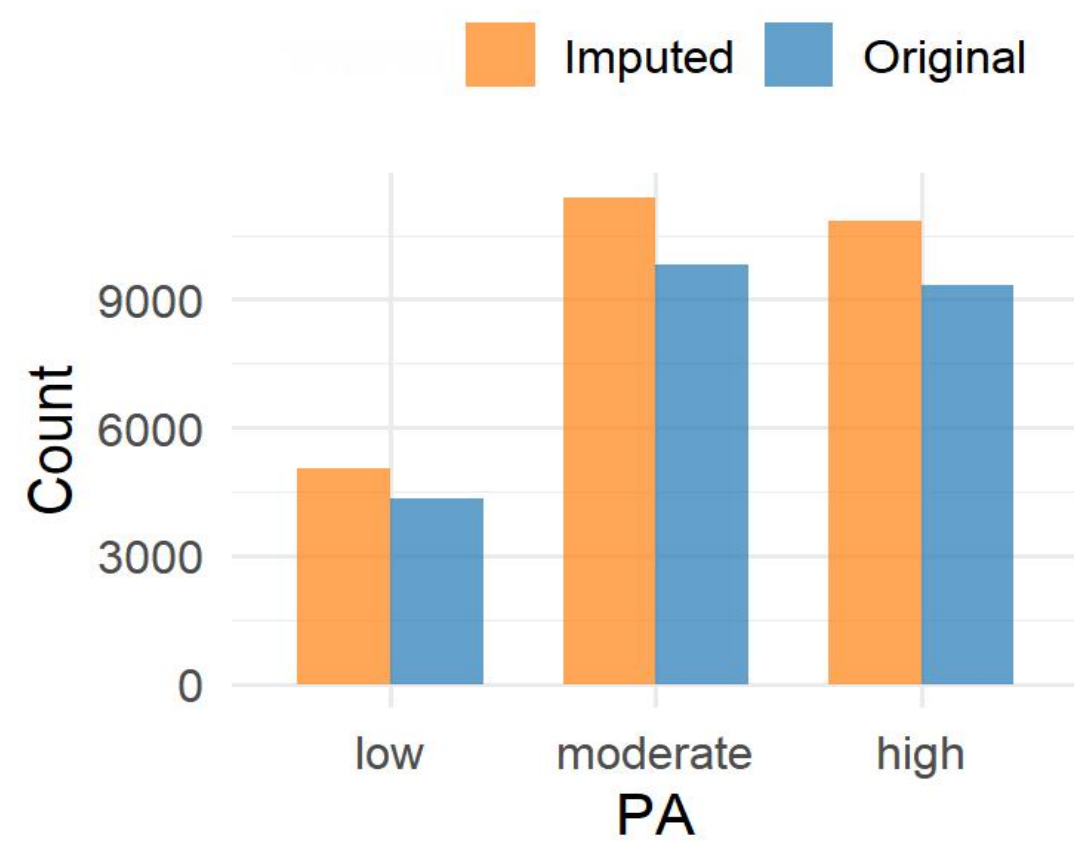

Supplementary Figure 1. Comparison of variable distribution before and after multiple imputation.

The figure summarizes the imputation results for the missing variable physical activity (PA), comparing data distributions pre- and post-imputation. PA, with a missing rate of 14.50% (5,495 of 37,886 participants), was imputed using multiple imputation by chained equations (MICE) with an ordinal logistic regression model. The imputation model included all other baseline characteristics as predictors, including age, sex, ethnicity, Townsend Deprivation Index, body mass index, body surface area, systolic blood pressure, diastolic blood pressure, heart rate, total cholesterol, high-density lipoprotein cholesterol, alcohol consumption status, smoking status and diabetes status. The results indicated that the overall data structure remained largely consistent before and after imputation.

A

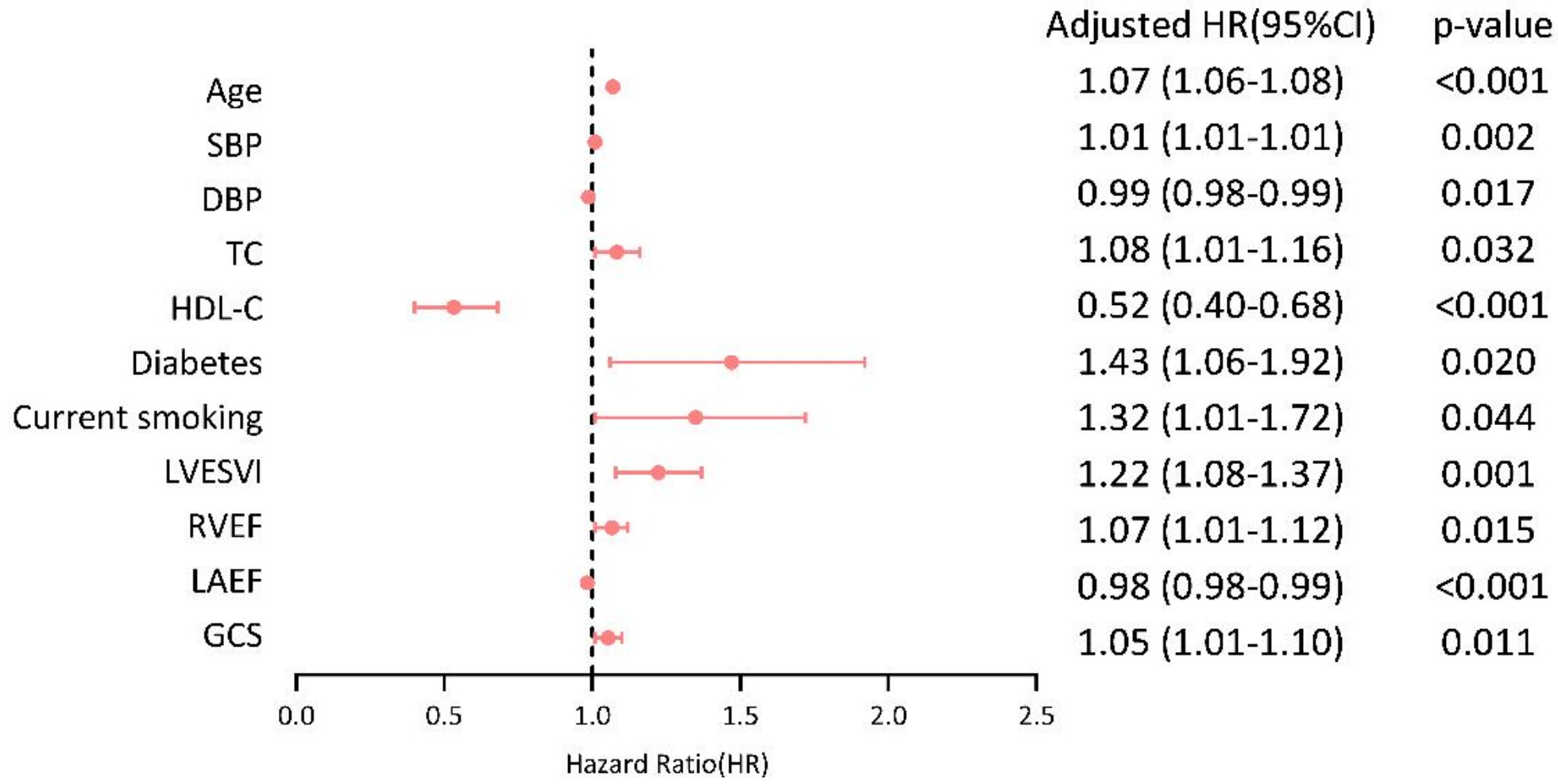
Adjusted HR(95%CI)
p-value
Age
1.07 (1.06-1.08)
<0.001
SBP
1.01 (1.01-1.01)
0.002
DBP
0.99 (0.98-0.99)
0.017
TC
1.08 (1.01-1.16)
0.032
HDL-C
0.52 (0.40-0.68)
<0.001
Diabetes
1.43 (1.06-1.92)
0.020
Current smoking
1.32 (1.01-1.72)
0.044
LVESVI
1.22 (1.08-1.37)
0.001
RVEF
1.07 (1.01-1.12)
0.015
LAEF
0.98 (0.98-0.99)
<0.001
GCS
1.05 (1.01-1.10)
0.011
0.0
0.5
1.0
1.5
2.0
2.5
Hazard Ratio(HR)

•

B

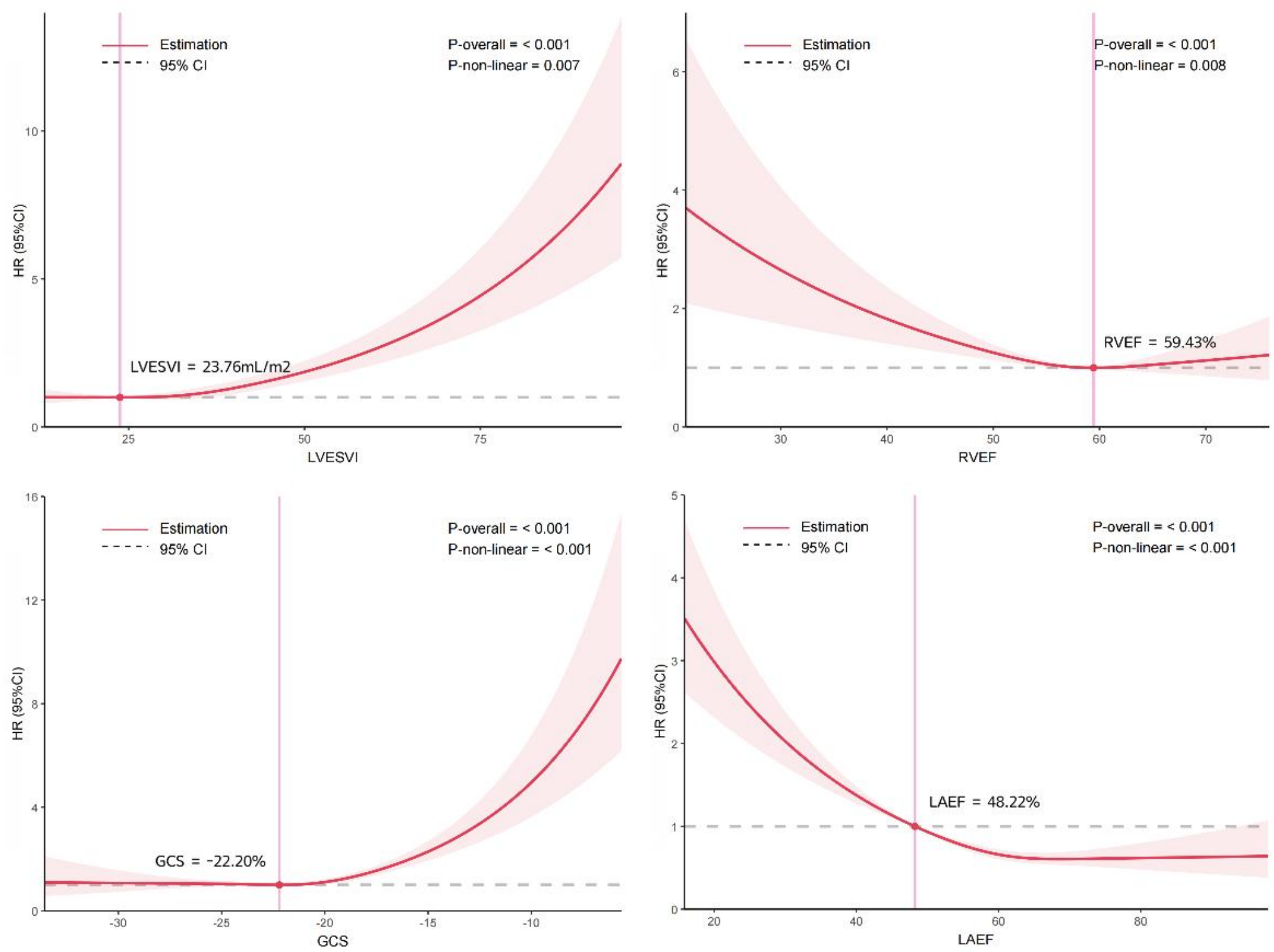
Estimation
95% CI
P-overall = < 0.001
P-non-linear = 0.007
LVESVI = 23.76mL/m2
HR (95%CI)
LVESVI
P-overall = < 0.001
P-non-linear = 0.008
RVEF = 59.43%
RVEF
P-overall = < 0.001
P-non-linear = < 0.001
GCS = -22.20%
GCS
P-overall = < 0.001
P-non-linear = < 0.001
LAEF = 48.22%
LAEF

•

•

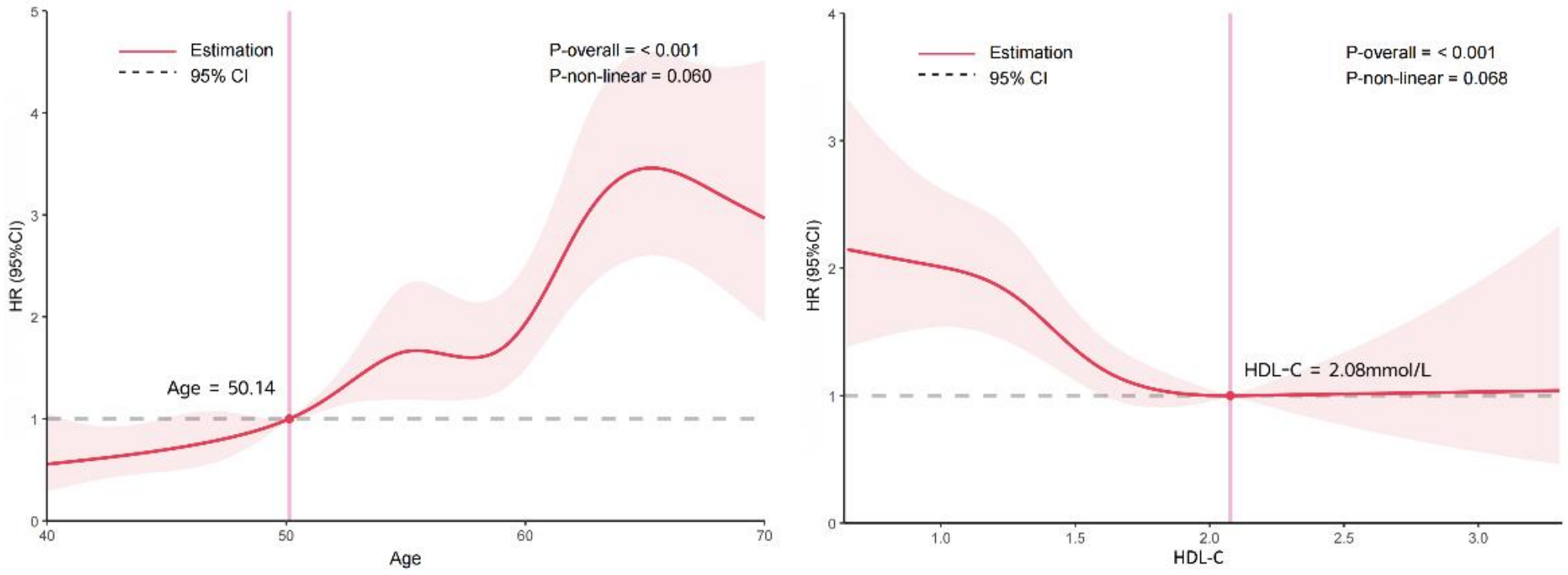

-
-

Supplementary Figure 2. Independent risk factors for MACE and dose-response relationship.
(A) Multivariable Cox regression analysis was performed to identify independent risk factors for MACE. Continuous covariates are standardized and hazard ratios reflect the change in cardiovascular risk per one-standard-deviation increase. Categorical covariates are converted into dummy variables. Only variables with statistical significance are presented. For the complete dataset, please contact the corresponding author (email: gaomedic@mail.xjtu.edu.cn).
(B) Dose-response relationships between independent risk factors and MACE were analyzed using RCS, adjusting for clinical covariates (sex, age, ethnicity, Townsend Deprivation Index, body mass index, body surface area, SBP, DBP, heart rate, diabetes, physical activity, alcohol consumption and smoking status, TC and HDL-C).
CI, confidence interval; DBP, diastolic blood pressure; GCS, global circumferential strain; HDL-C, high-density lipoprotein cholesterol; HR, hazard ratio; LAEF, left atrial ejection fraction; LVESVI, left ventricular end-systolic volume index; MACE, major adverse cardiovascular events; RCS, restricted cubic splines; RVEF, right ventricular ejection fraction; SBP, systolic blood pressure; TC, total cholesterol.

A

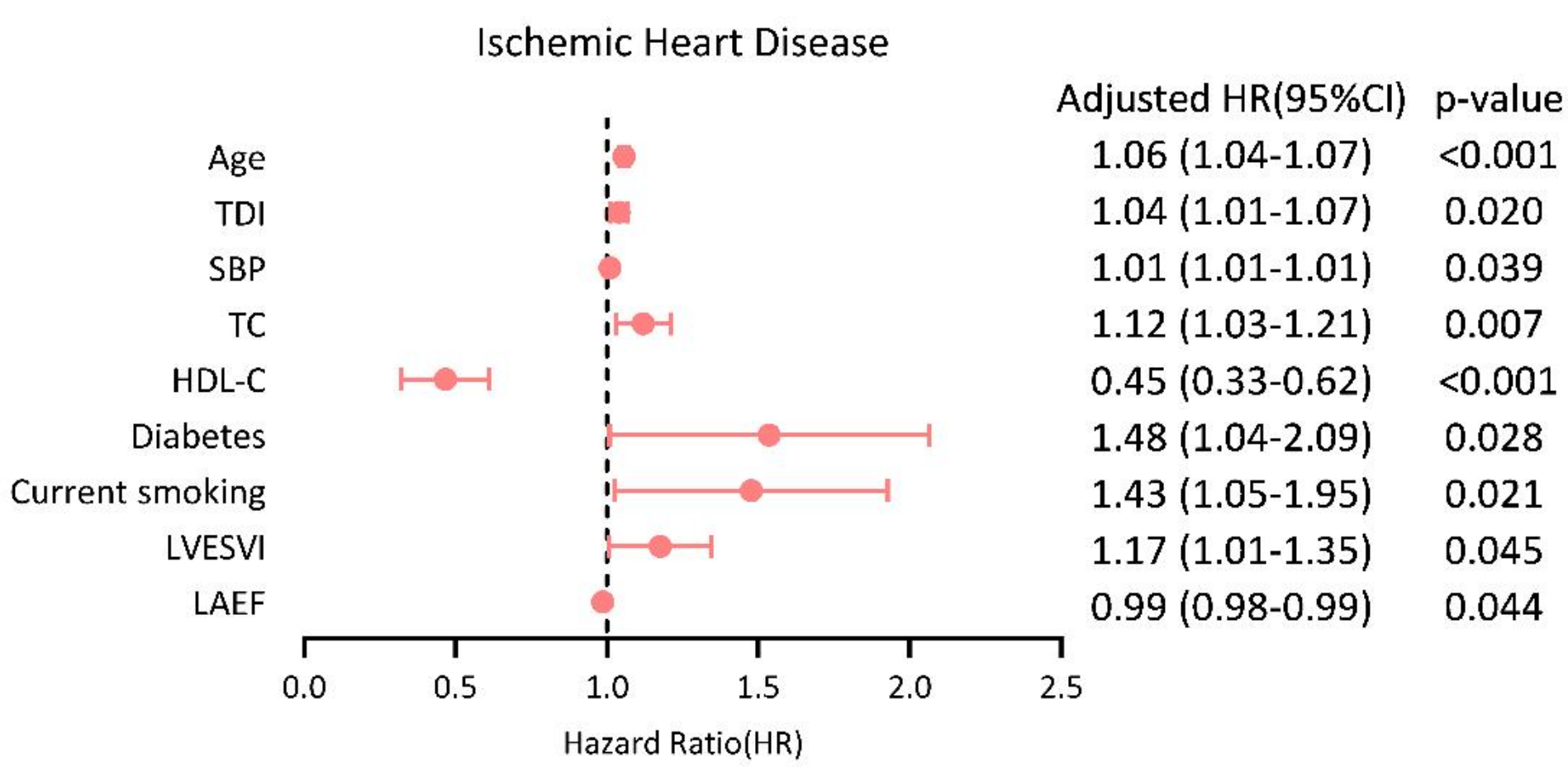

B

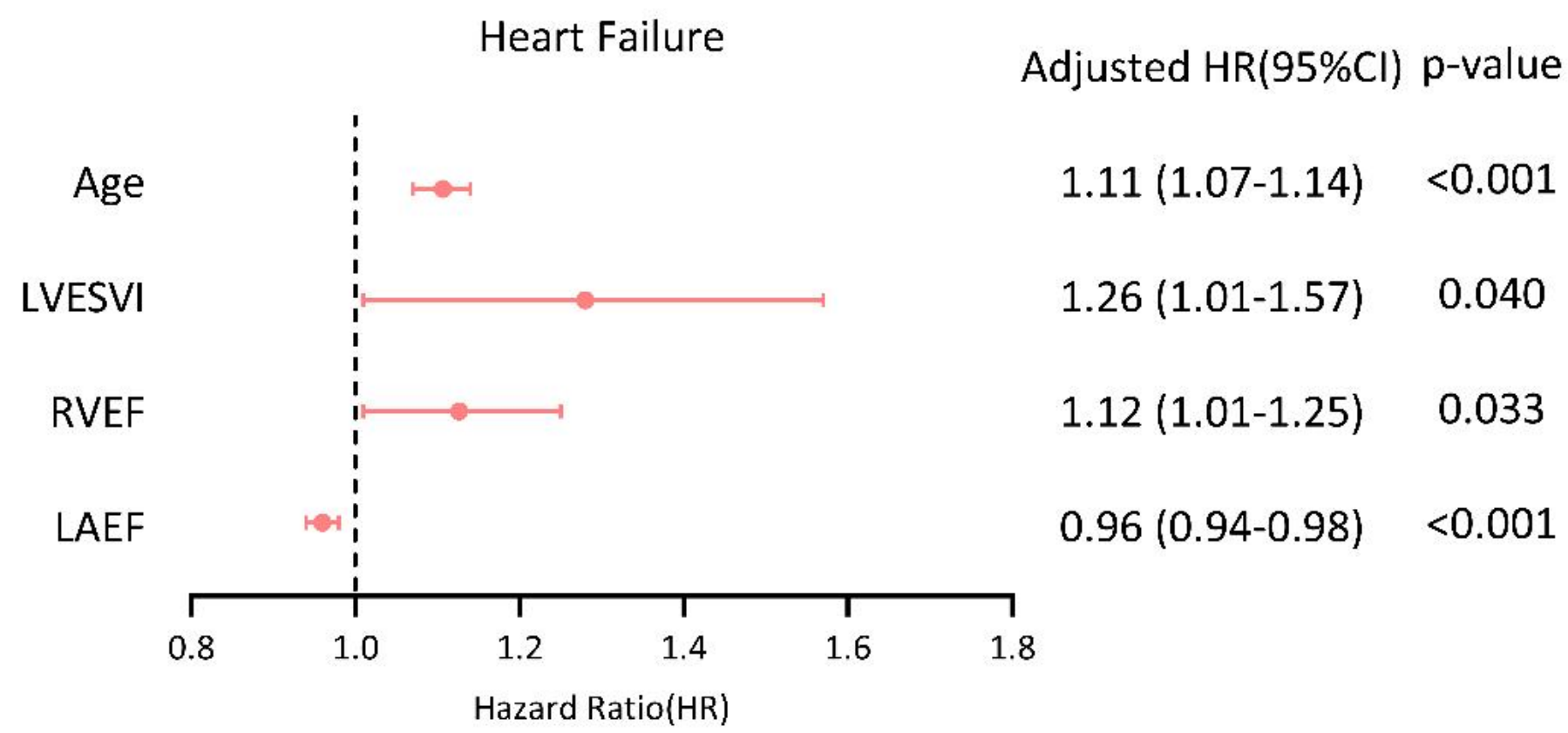

C

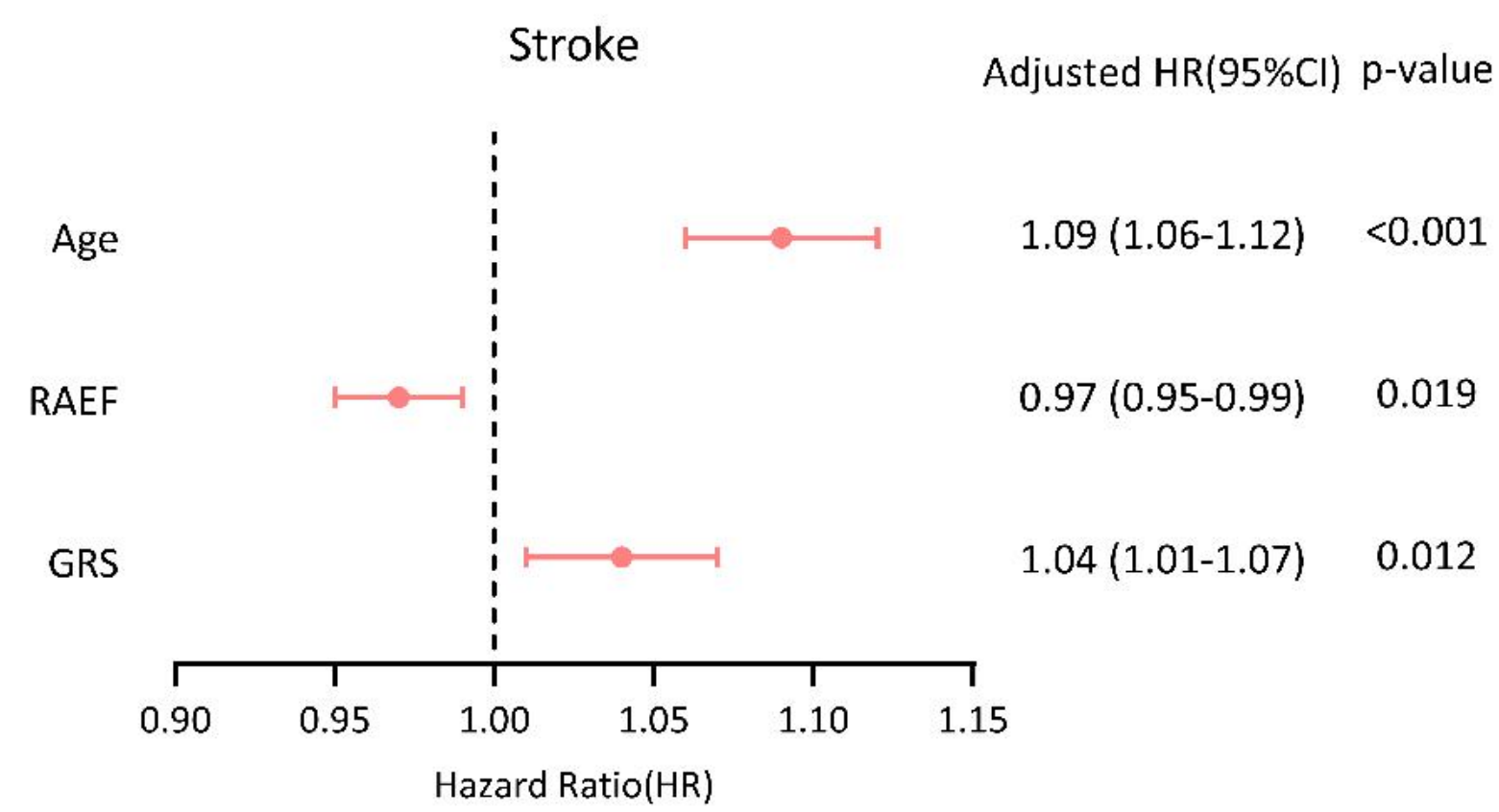

Supplementary Figure 3. Independent risk factors for specific MACE outcomes.

Multivariable Cox regression analysis was performed to identify independent risk factors for ischemic heart disease (A), heart failure (B) and stroke (C). Continuous covariates are standardized and hazard ratios reflect the change in cardiovascular risk per one-standard-deviation increase. Categorical covariates are converted into dummy variables. Only variables with statistical significance are presented. For the complete dataset, please contact the corresponding author (email: gaomedic@mail.xjtu.edu.cn).

GRS, global radial strain; HDL-C, high-density lipoprotein cholesterol; LAEF, left atrial ejection fraction; LVESVI, left ventricular end-systolic volume index; RAEF, right atrial ejection fraction; RVEF, right ventricular ejection fraction; SBP, systolic blood pressure; TC, total cholesterol; TDI, Thomson Deprivation Index.

A

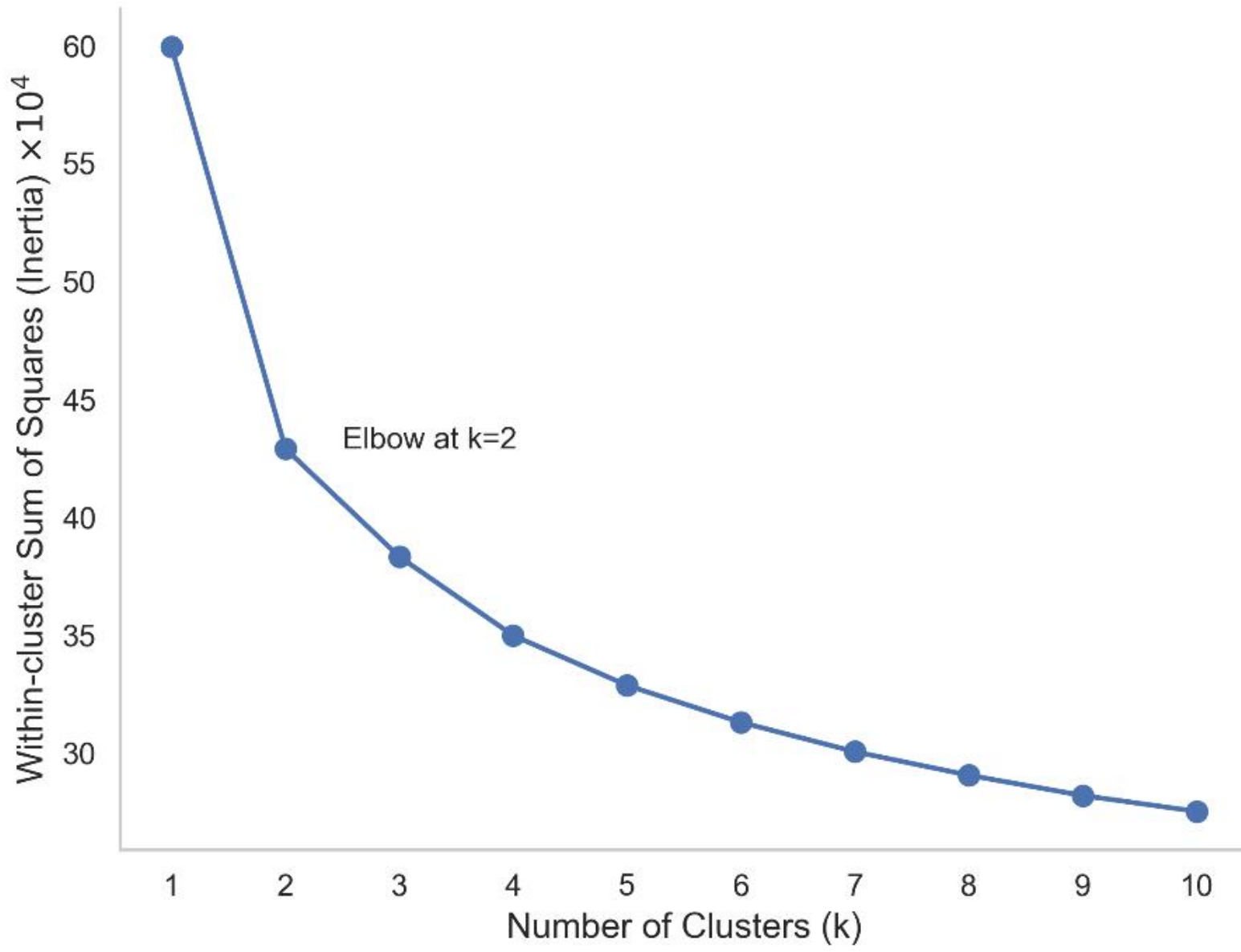

B

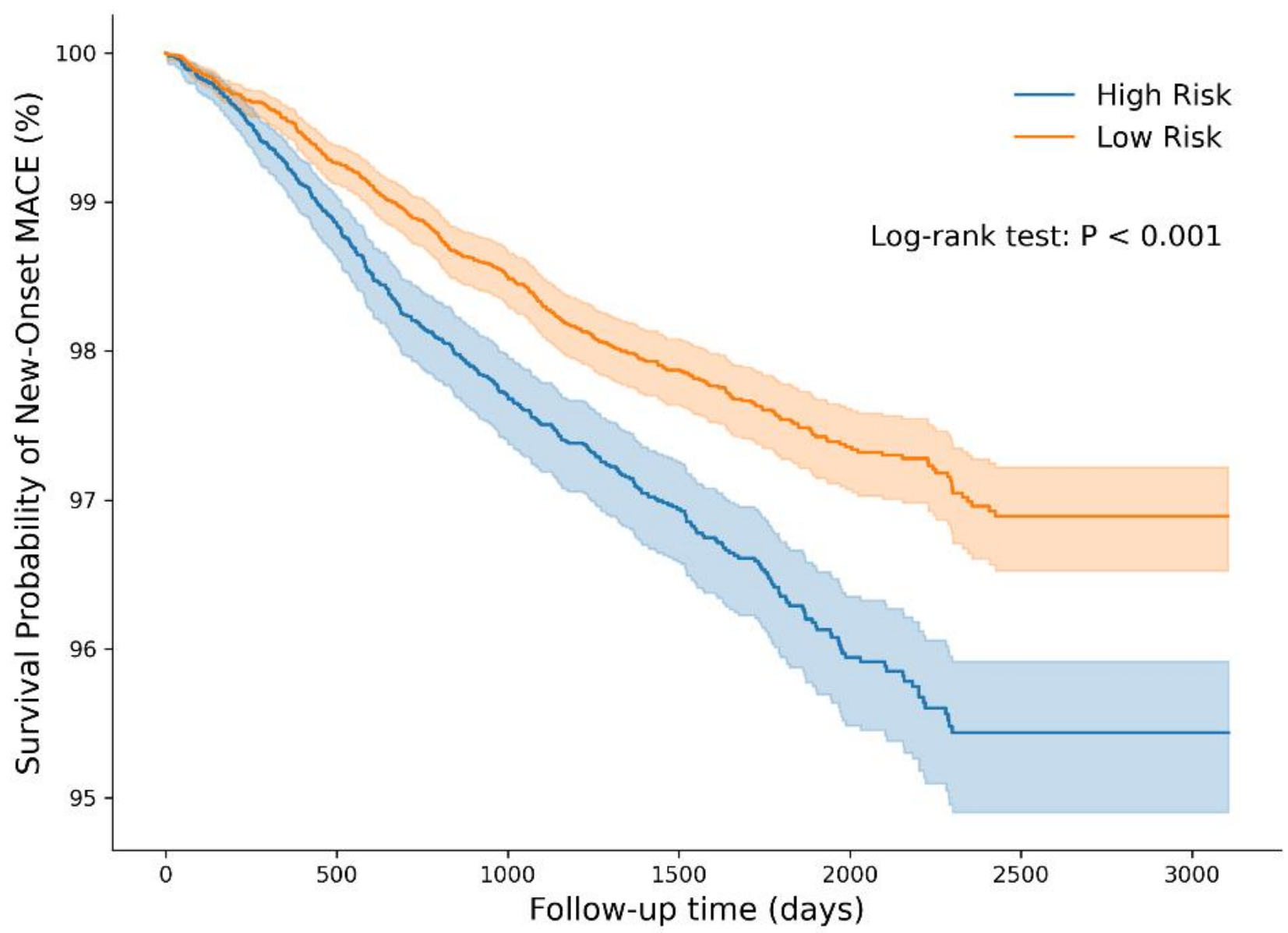

•

Supplementary Figure 4. Unsupervised clustering and survival analysis based on CMR-derived phenotypes.

(A) Unsupervised clustering was performed using 22 CMR metrics. The optimal number of clusters (k = 2) was determined using the elbow method, with a silhouette score of 0.42 indicating moderate-to-good cluster stability (High-Risk cluster: n=10,481; Low-Risk cluster: n=16,773).

(B) Kaplan-Meier survival analysis compares the risk of MACE between the two clusters, with statistical significance evaluated by the log-rank test.

CMR, cardiac magnetic resonance; MACE, major adverse cardiovascular events.

A

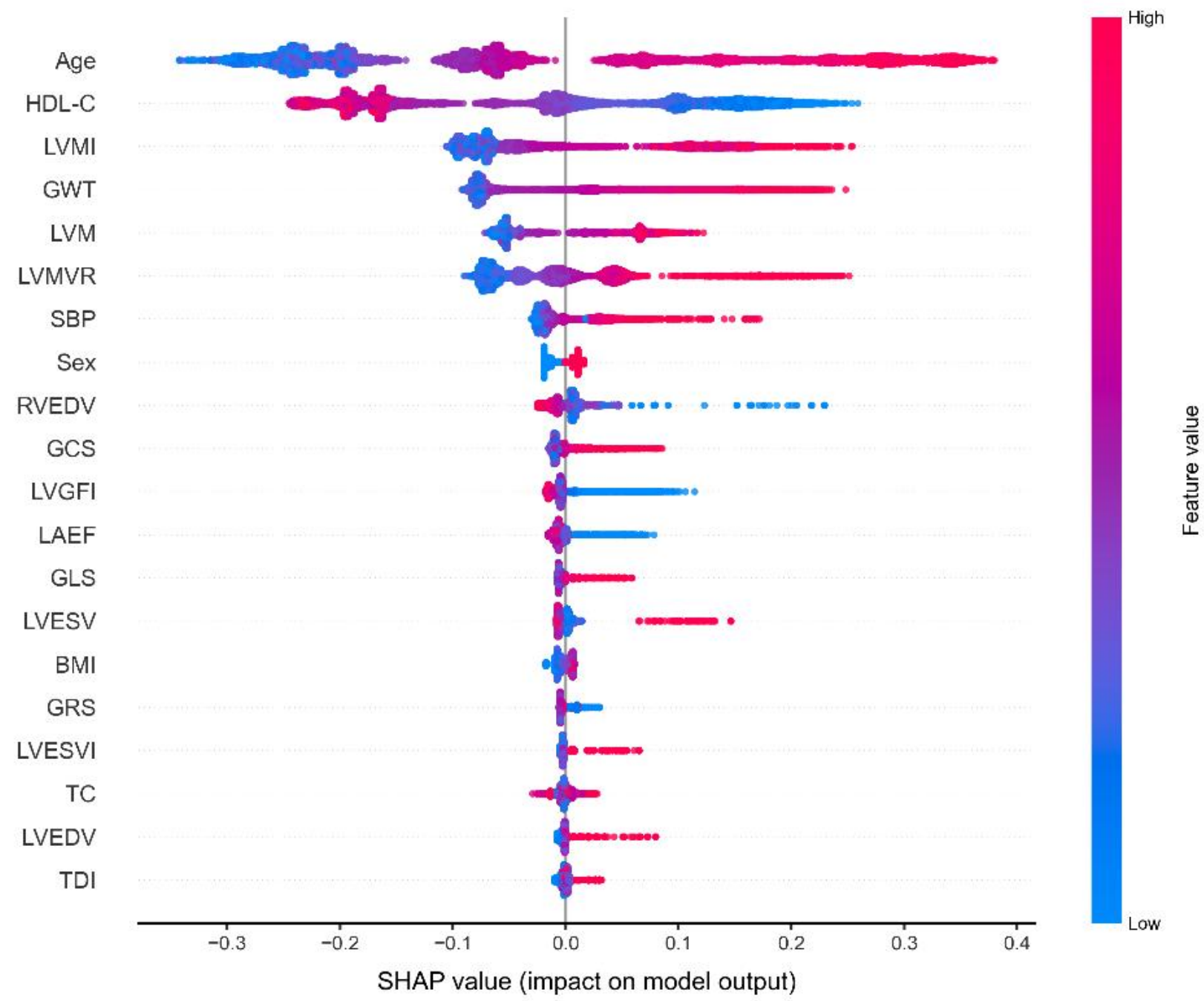
Age
HDL-C
LVMI
GWT
LVM
LVMVR
SBP
Sex
RVEDV
GCS
LVGFI
LAEF
GLS
LVESV
BMI
GRS
LVESVI
TC
LVEDV
TDI
−0.3
−0.2
−0.1
0.0
0.1
0.2
0.3
0.4
SHAP value (impact on model output)
High
Feature value
Low

B

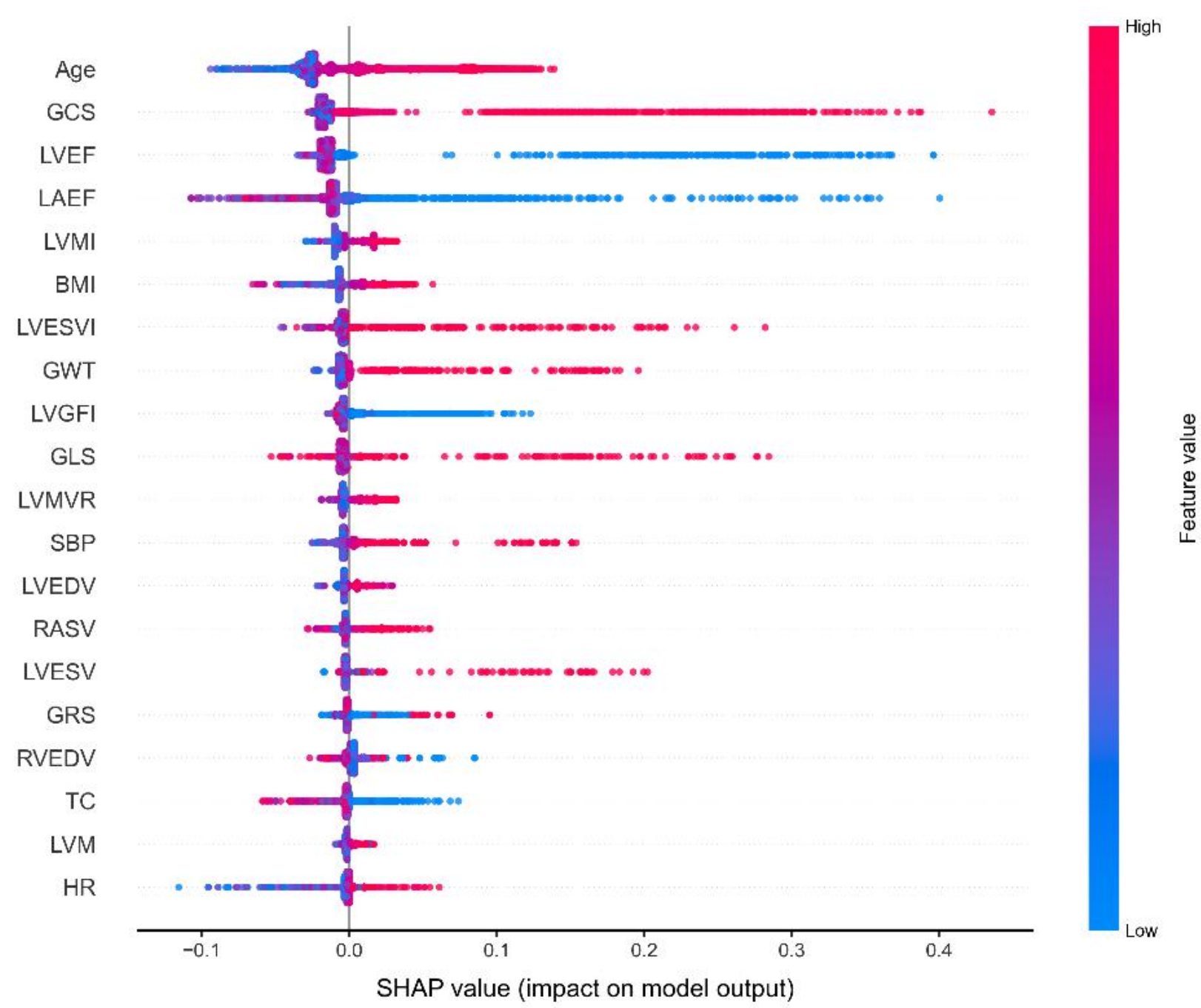
Age
GCS
LVEF
LAEF
LVMI
BMI
LVESVI
GWT
LVGFI
GLS
LVMVR
SBP
LVEDV
RASV
LVESV
GRS
RVEDV
TC
LVM
HR
−0.1
0.0
0.1
0.2
0.3
0.4
SHAP value (impact on model output)
High
Feature value
Low

C

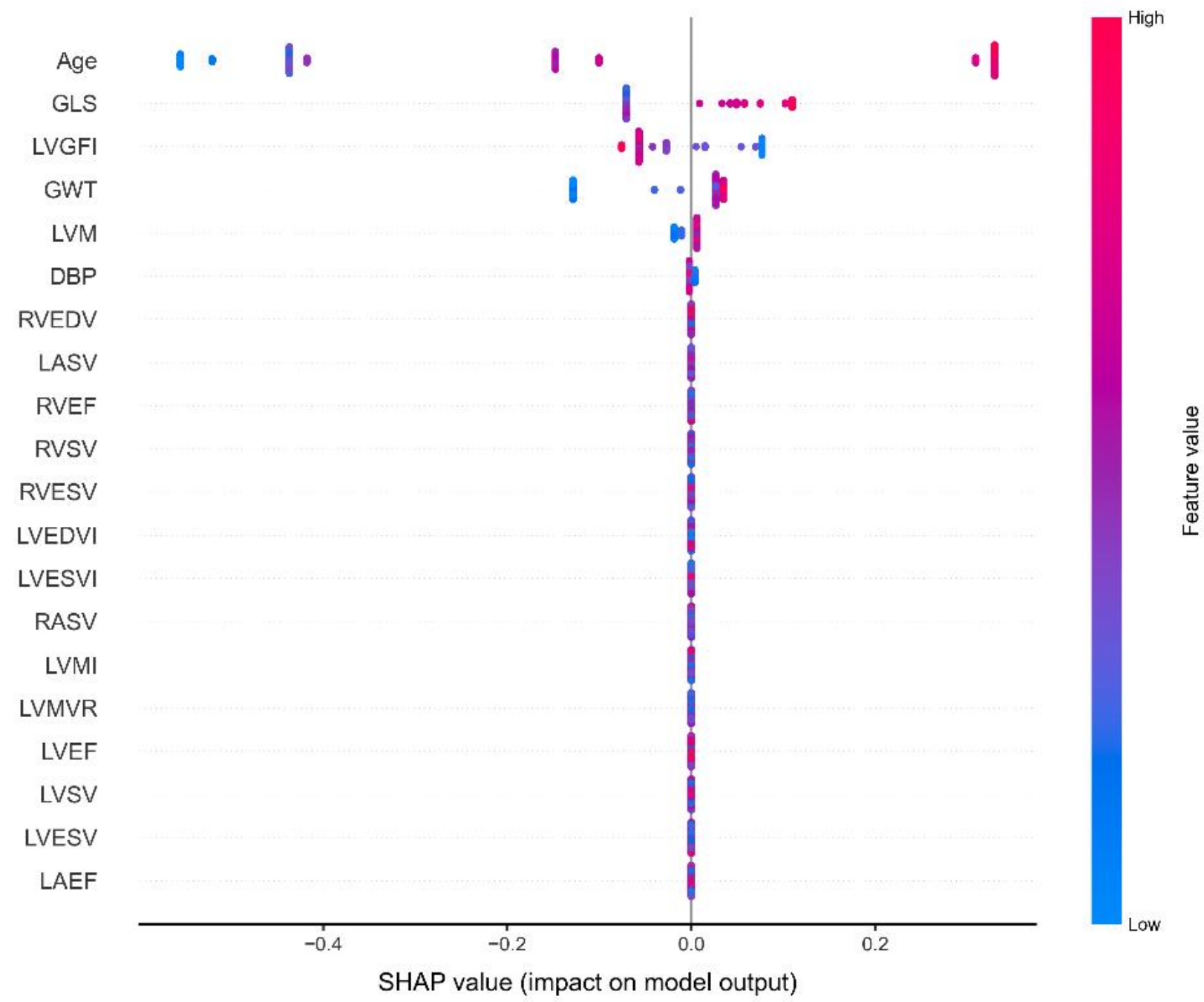

Supplementary Figure 5. SHAP ranking of the top 20 predictive features for specific MACE outcomes in CMR-enhanced models.

SHAP summary plots rank the top 20 features influencing predictors for ischemic heart disease (A), heart failure (B) and stroke (C). Features are ordered by importance along the y-axis. Each dot represents an individual patient, with color indicating feature value (blue = lower, red = higher). The x-axis reflects SHAP value, demonstrating the feature's impact on model output. Higher SHAP values indicate a greater likelihood of developing cardiovascular diseases.

BMI, body mass index; CMR, cardiac magnetic resonance; DBP, diastolic blood pressure; GCS, global circumferential strain; GLS, global longitudinal strain; GRS, global radial strain; GWT, global wall thickness; HDL-C, high-density lipoprotein cholesterol; HR, heart rate; LAEF, left atrial ejection fraction; LASV, left atrial stroke volume; LV, left ventricular; LVEF, left ventricular ejection fraction; LVEDV, left ventricular end-diastolic volume; LVEDVI, left ventricular end-diastolic volume index; LVESV, left ventricular end-systolic volume; LVESVI, left ventricular end-systolic volume index; LVGFI, left ventricular global function index; LVM, left ventricular mass; LVMVR, left ventricular mass-to-volume ratio; LVMI, left ventricular mass index; LVSV, left ventricular stroke volume; MACE, major adverse cardiovascular events; RASV, right atrial stroke volume; RV, right ventricular; RVEDV, right ventricular end-diastolic volume; RVEF, right ventricular ejection fraction; RVESV, right ventricular end-systolic volume; RVSV, right ventricular stroke volume; SBP, systolic blood pressure; SHAP, Shapley Additive Explanations; TC, total cholesterol; TDI, Thomson Deprivation Index.

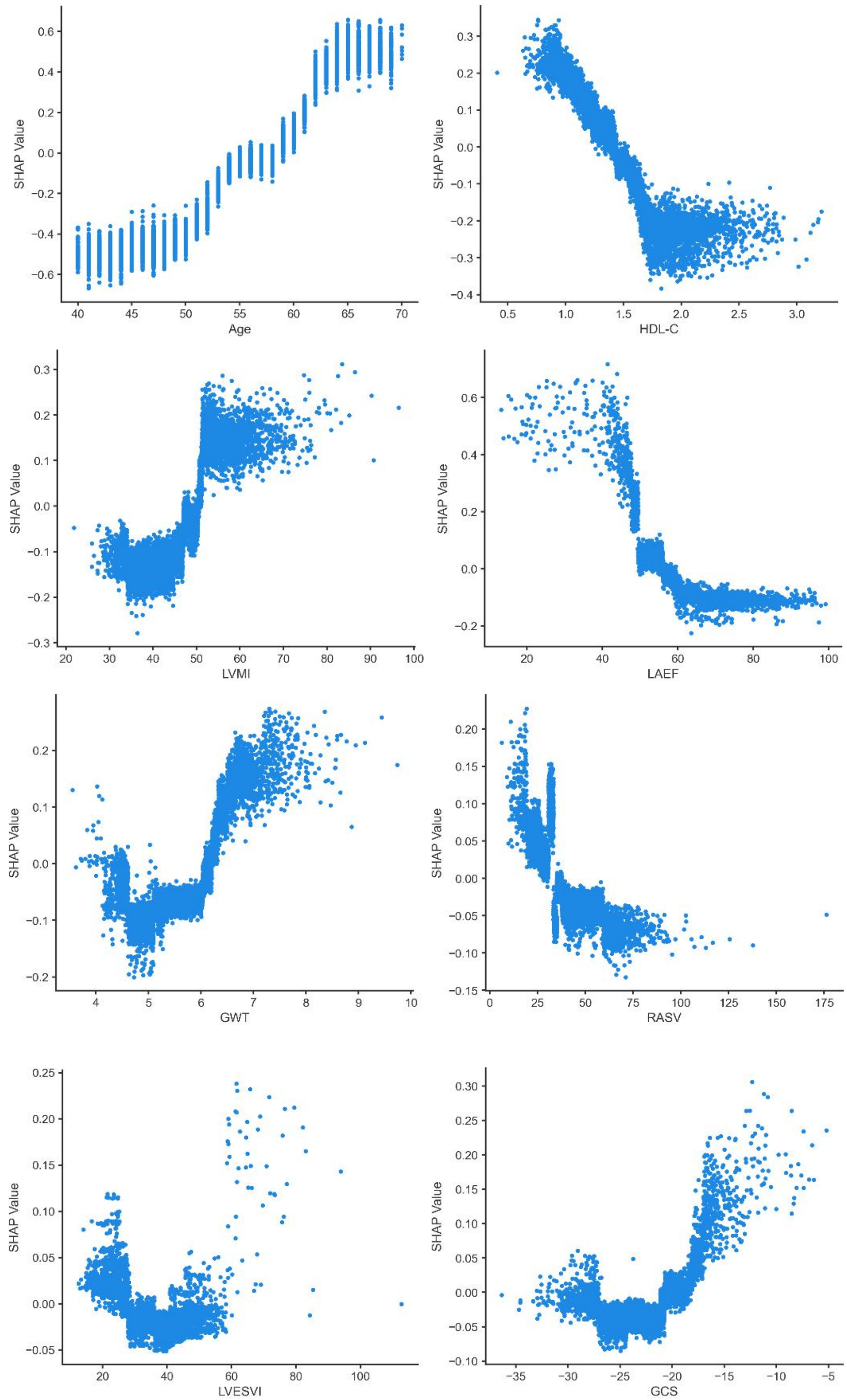

Supplementary Figure 6. SHAP dependence plots illustrating the relationship between predictors and MACE risk.

Each dot represents one participant, with the x-axis showing the actual feature value and the y-axis indicating the corresponding SHAP value, reflecting the feature's contribution to the predicted risk of MACE. Positive SHAP values indicate an increased probability of MACE occurrence. Dot color

denotes the relative feature value (from low to high), enabling visualization of nonlinear or threshold effects.
CMR, cardiac magnetic resonance; GCS, global circumferential strain; GWT, global wall thickness; HDL-C, high-density lipoprotein cholesterol; LAEF, left atrial ejection fraction; LVESVI, left ventricular end-systolic volume index; LVMI, LV mass index; MACE, major adverse cardiovascular events; RASV, right atrial stroke volume; SHAP, Shapley Additive Explanations.

A

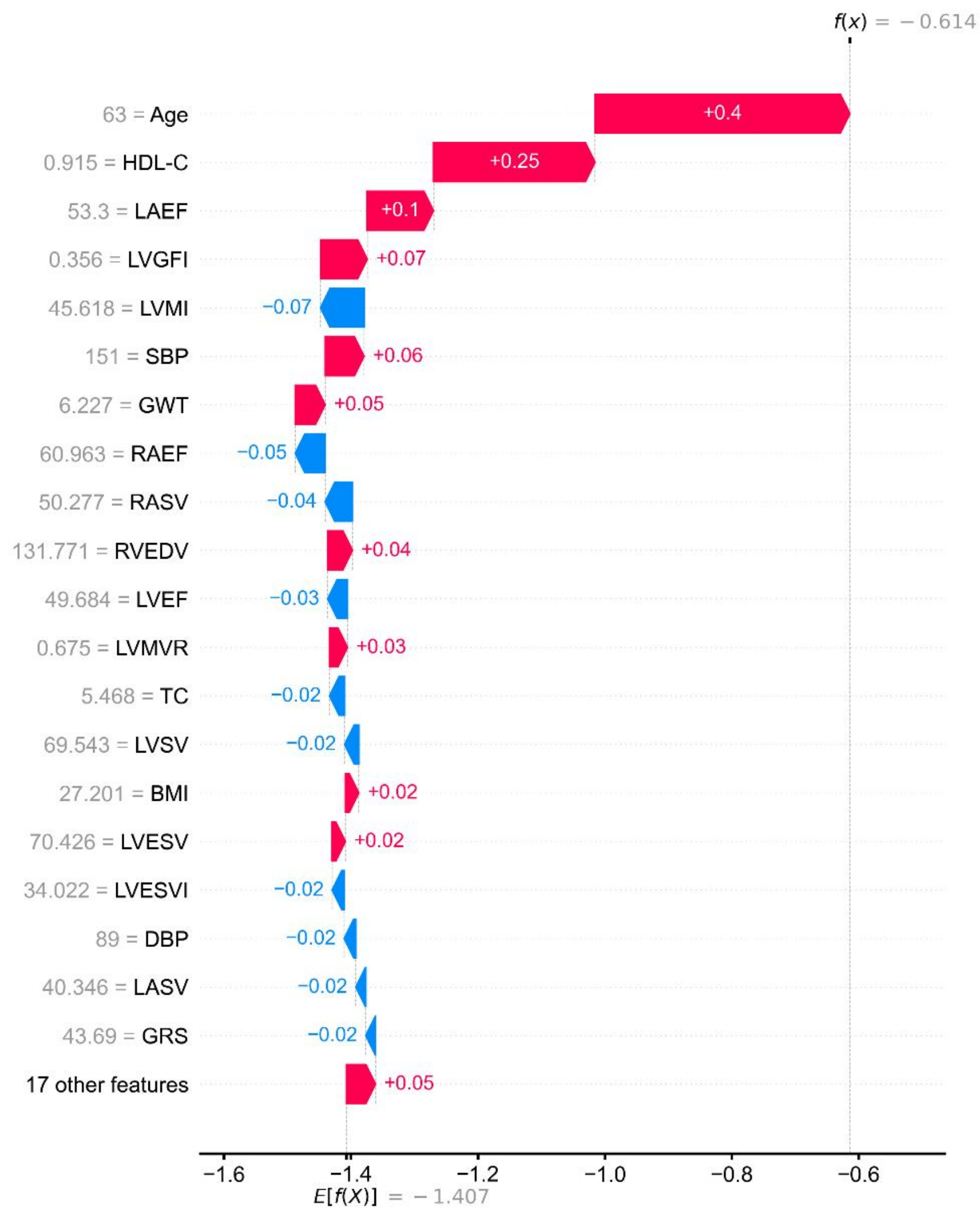

B

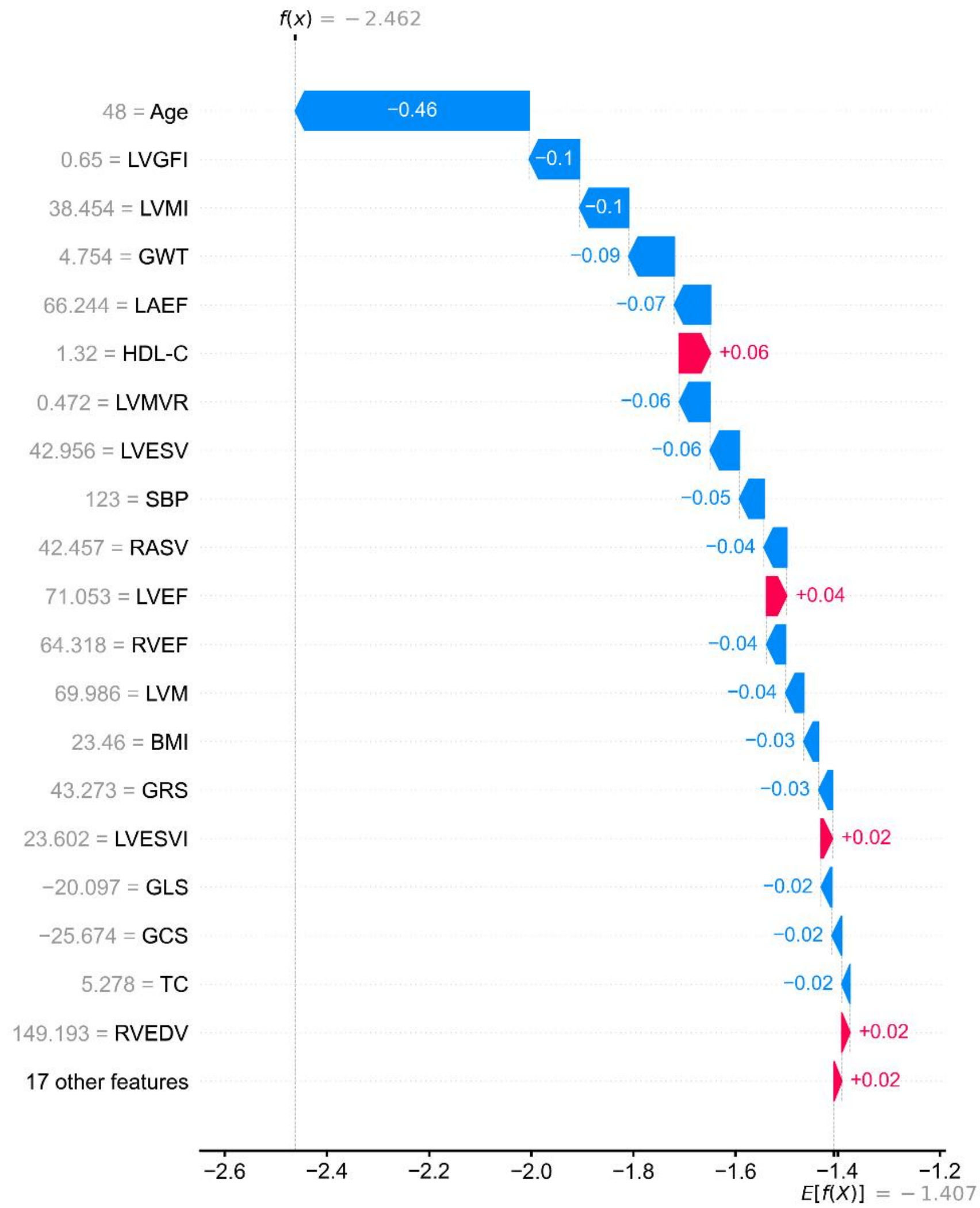

Supplementary Figure 7. Waterfall plots of individual MACE risk profiles.
The two plots present two individual cases, respectively illustrating one predicted to develop MACE (A) and another predicted not to (B), with predictions aligning with actual clinical outcomes. Waterfall plots show the top 20 most important features for predicting MACE risk at the individual level. The x-axis reflects SHAP value. The y-axis indicates the specific feature values of each individual. Positive (red) and negative (blue) SHAP values indicate the contribution of each feature to increasing or decreasing predicted risk, respectively. The total score above represents the overall risk prediction. If the total score exceeds the baseline value at the bottom (E[f(x) = -1.407]), the model predicts the occurrence of MACE. Numbers within bars denote SHAP value for each feature, quantifying their impact on predicted MACE risk.
BMI, body mass index; DBP, diastolic blood pressure; GCS, global circumferential strain; GLS, global longitudinal strain; GRS, global radial strain; GWT, global wall thickness; HDL-C, high-density lipoprotein cholesterol; LASV, left atrial stroke volume; LAEF, left atrial ejection fraction; LVEF, left ventricular ejection fraction; LVM, left ventricular mass; LVMVR, left ventricular mass-to-volume ratio; LVESV, left ventricular end-systolic volume; LVESVI, left ventricular end-systolic volume index; LVGFI, left ventricular global function index; LVMI, left ventricular mass index; LVSV, left ventricular stroke volume; MACE, major adverse cardiovascular events; RAEF, right atrial ejection fraction; RASV, right atrial stroke volume; RVEDV, right ventricular end-diastolic

volume; RVEF, right ventricular ejection fraction; SBP, systolic blood pressure; SHAP, Shapley Additive Explanations; TC, total cholesterol.

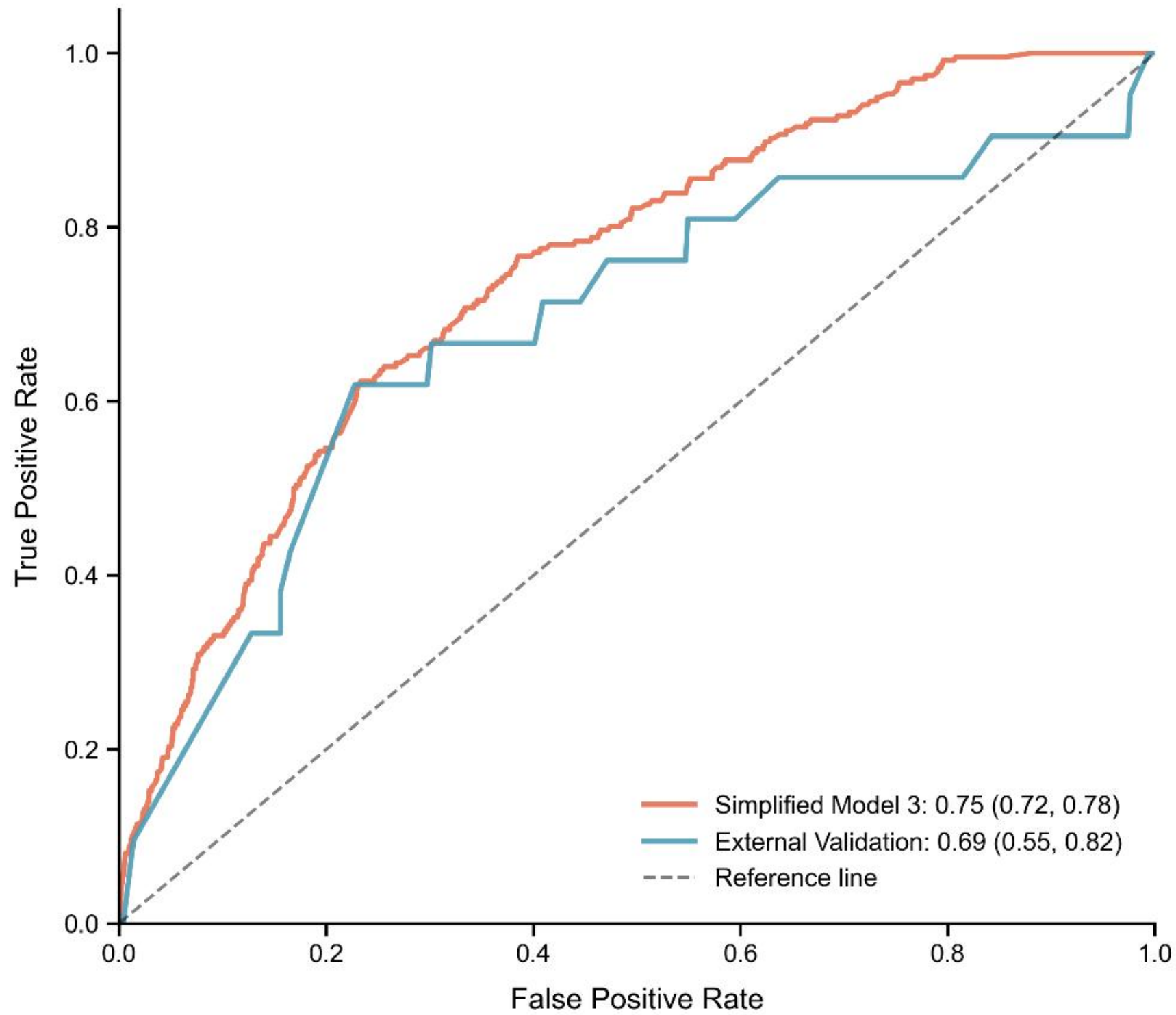

Supplementary Figure 8. ROC curves of the simplified model before removal of collinear variables and its external validation.

Exclusion of the non-echocardiographic predictor (right ventricular end-diastolic volume) and the highly collinear left ventricular end-diastolic volume (retained left ventricular end-diastolic volume index instead) did not materially affect the model's predictive performance, confirming the robustness of the final model comprising eight predictors: age, body surface area, left ventricular end-diastolic volume index, left ventricular end-systolic volume index, left ventricular ejection fraction, left ventricular mass index, left ventricular mass-to-volume ratio, and left ventricular global function index.

2.2 Supplementary Tables

Supplementary Table 1. Criteria for cohort selection and outcome identification (Field IDs and ICD-10 Codes) in UK Biobank and external validation cohort.

Outcome	UKBB data field(s)	Code
Heart failure	41270	I50, I500, I501, I509
	41280	
Ischemic heart disease	41270	I20, I200, I201, I208, I209, I21, I210, I211, I212, I213, I214, I219, I22, I220, I221,

	41280	I228, I229, I23, I230, I231, I232, I233, I235, I236, I238, I24, I240, I241, I248, I249, I25, I250, I251, I252, I253, I254, I255, I256, I258, I259
Stroke	41270<br>41280	I60, I600, I601, I602, I603, I604, I605, I606, I607, I608, I609, I61, I610, I611, I612, I613, I614, I615, I616, I618, I619, I62, I620, I621, I629, I63, I630, I631, I632, I633, I634, I635, I636, I638, I639, I64
Major adverse cardiovascular events	41270<br>41280	I50, I500, I501, I509, I20, I200, I201, I208, I209, I21, I210, I211, I212, I213, I214, I219, I22, I220, I221, I228, I229, I23, I230, I231, I232, I233, I235, I236, I238, I24, I240, I241, I248, I249, I25, I250, I251, I252, I253, I254, I255, I256, I258, I259, I60, I600, I601, I602, I603, I604, I605, I606, I607, I608, I609, I61, I610, I611, I612, I613, I614, I615, I616, I618, I619, I62, I620, I621, I629, I63, I630, I631, I632, I633, I634, I635, I636, I638, I639, I64
Diabetes	41270<br>41280	E10, E100, E101, E102, E103, E104, E105, E106, E107, E108, E109, E11, E110, E111, E112, E113, E114, E115, E116, E117, E118, E119
Death	4000	Date death occurred

Summary of cohort selection criteria and outcome definitions, including corresponding ICD-10 codes and UK Biobank field IDs.

ICD-10, International Classification of Diseases, 10th Revision.

Supplementary Table 2. Predictive performance of XGBOOST models for MACE outcomes.

Outcome	Model 1	Model 2	Model 3

MACE	0.61(0.58, 0.64)	0.63(0.59, 0.66)	0.76(0.73, 0.79)
IHD	0.59(0.55, 0.63)	0.59(0.55, 0.63)	0.71(0.67, 0.75)
HF	0.63(0.55, 0.71)	0.73(0.64, 0.80)	0.90(0.86, 0.94)
Stroke	0.61(0.53, 0.68)	0.62(0.54, 0.69)	0.75(0.69, 0.81)

Three sequential XGBOOST Models are evaluated by AUC across cardiovascular outcomes, including MACE, IHD, HF and stroke. The AUC reflects each model's discriminative ability, with 95% confidence intervals estimated using bootstrapping with 1,000 iterations.

AUC, area under the receiver operating characteristic curve; HF, heart failure; IHD, ischemic heart disease; MACE, major adverse cardiovascular events; XGBoost, Extreme Gradient Boosting.

Supplementary Table 3. Baseline characteristics of the external validation cohort stratified by MACE outcome.

**Baseline characteristics**	**All cohort (n=522)**	**MACE (n = 21)**	**MACE-free (n= 501)**	**P-value**
Age, year	51.97 ± 13.74	62.43 ± 10.90	51.53 ± 13.68	P < 0.001
BSA, m^2	1.72 ± 0.17	1.66 ± 0.16	1.73 ± 0.17	0.066
LVEF (%)	0.67 ± 0.05	0.67 ± 0.05	0.68 ± 0.05	0.770
LVEDVI (mL/m^2)	56.68 ± 10.95	63.97 ± 16.22	56.38 ± 10.58	0.046
LVESVI (mL/m^2)	20.70 ± 5.87	21.36 ± 8.57	20.68 ± 5.74	0.721
LVGFI (%)	0.30 ± 0.05	0.32 ± 0.04	0.30 ± 0.05	0.051
LVMI (g/m^2)	86.19 ± 14.71	98.43 ± 23.44	85.68 ± 14.03	0.022
LVMVR (g/mL)	1.53 ± 0.16	1.56 ± 0.20	1.53 ± 0.15	0.580

Baseline characteristics were presented as means (standard deviation) for continuous variables and counts (percentages) for categorical variables, compared using t-tests and chi-square tests respectively.

BSA, body surface area; LVEF, left ventricular ejection fraction; LVEDVI, left ventricular end-diastolic volume index; LVESVI, left ventricular end-systolic volume index; LVGFI, left ventricular global function index; LVMI, left ventricular mass index; LVMVR, left ventricular mass-to-volume ratio; MACE, major adverse cardiovascular events.

3 Supplemental References

1. D'Agostino, R. B., Sr *et al.* General cardiovascular risk profile for use in primary care: the Framingham Heart Study. *Circulation* **117**, 743–53 (2008).

2. Woodward, M., Brindle, P., Tunstall-Pedoe, H. & SIGN group on risk estimation. Adding social deprivation and family history to cardiovascular risk assessment: the ASSIGN score from the

Scottish Heart Health Extended Cohort (SHHEC). *Heart* **93**, 172–6 (2007).

3. Victor, R. G. *et al.* The Dallas Heart Study: a population-based probability sample for the multidisciplinary study of ethnic differences in cardiovascular health. *Am J Cardiol* **93**, 1473–80 (2004).

4. Arsenault, B. J., Boekholdt, S. M. & Kastelein, J. J. P. Lipid parameters for measuring risk of cardiovascular disease. *Nat Rev Cardiol* **8**, 197–206 (2011).

5. Che, B. *et al.* Triglyceride-glucose index and triglyceride to high-density lipoprotein cholesterol ratio as potential cardiovascular disease risk factors: an analysis of UK biobank data. *Cardiovasc Diabetol* **22**, 34 (2023).

6. Bai, W. *et al.* Automated cardiovascular magnetic resonance image analysis with fully convolutional networks. *J Cardiovasc Magn Reson* **20**, 65 (2018).

7. Bai, W. *et al.* A population-based phenome-wide association study of cardiac and aortic structure and function. *Nat Med* **26**, 1654–1662 (2020).

8. Wang, S. *et al.* AI Based CMR Assessment of Biventricular Function: Clinical Significance of Intervendor Variability and Measurement Errors. *JACC Cardiovasc Imaging* **15**, 413–427 (2022).

9. Lima, J. A. C. & Venkatesh, B. A. Building Confidence in AI-Interpreted CMR. *JACC Cardiovasc Imaging* **15**, 428–430 (2022).

10. Petersen, S. E. *et al.* UK Biobank's cardiovascular magnetic resonance protocol. *J Cardiovasc Magn Reson* **18**, 8 (2016).

11. Liao, Y., Cooper, R. S., Durazo-Arvizu, R., Mensah, G. A. & Ghali, J. K. Prediction of mortality risk by different methods of indexation for left ventricular mass. *J Am Coll Cardiol* **29**, 641–7 (1997).

12. Xu, H., Chen, C., Lu, Z. X., & Nie, Z. rcssci: An R package for visualization of restricted cubic spline. *Med Research*, **1**, 201–206 (2025).

13. Capron, T. *et al.* Cardiac magnetic resonance assessment of left ventricular dilatation in chronic severe left-sided regurgitations: comparison with standard echocardiography. *Diagn Interv Imaging* **101**, 657–665 (2020).

14. Zhao, L., Lu, A., Tian, J., Huang, J. & Ma, X. Effects of Different LVEF Assessed by Echocardiography and CMR on the Diagnosis and Therapeutic Decisions of Cardiovascular Diseases. *Front Physiol* **11**, 679 (2020).

15. Sun, X., & Xu, W. Fast implementation of DeLong's algorithm for comparing the areas under correlated receiver operating characteristic curves. IEEE Signal Processing Letters, 21, 1389–1393 (2014).
